# Biosynthesis Regulation of Secondary Metabolite Production in *Fusarium* Fungi

**DOI:** 10.3390/jof11110820

**Published:** 2025-11-20

**Authors:** Prosper Amuzu, Xiaoqian Pan, Xuwen Hou, Yu Li, Jiahang Sun, Yujun Huang, Pengfei Wang, Liyao Liu, Daowan Lai, Ligang Zhou

**Affiliations:** Department of Plant Pathology, College of Plant Protection, China Agricultural University, Beijing 100193, China; amuzuprosper07@gmail.com (P.A.); xiaoqianpan@cau.edu.cn (X.P.); xwhou@cau.edu.cn (X.H.); yuli@cau.edu.cn (Y.L.); jiahangsun@cau.edu.cn (J.S.); yujunhuang@cau.edu.cn (Y.H.); pengfeiwang@cau.edu.cn (P.W.); lyliu@cau.edu.cn (L.L.); dwlai@cau.edu.cn (D.L.)

**Keywords:** *Fusarium* fungi, secondary metabolites, biosynthesis regulation, environmental factors, transcriptional factors, epigenetic regulation, signal transduction regulation

## Abstract

*Fusarium* fungi are prolific producers of a wide array of structurally and functionally diverse secondary metabolites (SMs), ranging from harmful mycotoxins to beneficial phytohormones and medicines. Many of these compounds show significant promise for use as agrochemicals, pharmaceuticals and food additives. The biosynthesis of these SMs in *Fusarium* fungi is strictly regulated by a complex network composed of various regulatory components. This review highlights recent advances in understanding how secondary metabolism in *Fusarium* fungi is regulated at various levels, particularly through the regulation of environmental factors (e.g., light, temperature, pH, carbon, and nitrogen sources), global and pathway-specific transcriptional factors (e.g., LaeA, LaeB, AreA, Tri6, and ZEB2), epigenetic modifications (e.g., histone acetylation and methylation, DNA and RNA modifications), and signal transduction pathways (e.g., cAMP, TOR, and MAPK pathways). Furthermore, the biological significances and potential applications of some metabolites (e.g., beauvericin, bikaverin, gibberellins, fumonisins, fusaric acid, and trichothecenes) produced by *Fusarium* fungi were discussed. Biosynthesis regulation on SM production offers a powerful approach to either unlock silent or cryptic biosynthetic gene clusters (BGCs) for the discovery of new SMs, to boost the yiled of low-abundance beneficial metabolites, or suppress specific BGCs to eliminate the production of toxic compounds in *Fusarium* fungi.

## 1. Introduction

*Fusarium* fungi are extremely widespread and ubiquitous in terrestrial and marine environments. They belong to the Nectriaceae, Hypocreales, Sordariomycetes of ascomycetous fungi [1,2]. *Fusarium* fungi include marine-derived, soil-derived, endophytic, and pathogenic species. Since 1809, when Link initially described and defined *F. roseum* from Malavaceous plants, more than 1000 *Fusarium* species have been described in this genus [3,4].

Many *Fusarium* species can produce structurally diverse secondary metabolites (SMs) to provide protection and survival for themselves in the environment. Furthermore, these metabolites have multiple biological activities including antimicrobial, cytotoxic, antioxidant, nematocidal, and plant growth regulatory activities. On the one hand, *Fusarium* fungi are the producers of mycotoxins, but on the other hand, they are an important source of bioactive compounds. This has attracted increasing attention for the study of bioactive metabolites of *Fusarium* fungi [5,6]. *Fusarium*-derived secondary metabolites mainly include polyketides, terpenoids, nitrogen-containing compounds, phenolics, and steroids [7,8,9]. Some of these metabolites (e.g., deoxynivalenol, zearalenone, fusaric acid, fusariotoxin T2 and fumonisin B1) are called mycotoxins or phytotoxins due to the toxicity on animals or plants, causing their diseases [10,11,12]. However, most *Fusarium* species can produce valuable bioactive SMs, demonstrating potential applications in the pharmaceutical, agricultural and food industries. This makes them a treasure trove of bioactive compounds [6,8,13,14].

In order to either reveal additional bioactive SMs, increase the yield of useful known SMs, or inhibit toxic metabolite production, the regulation of biosynthetic pathways in *Fusarium* fungi offered a promising strategy to enhance the production of low-yield valuable SMs, suppress toxic compounds, and uncover novel bioactive molecules. Biosynthesis regulation mainly includes environmental factor regulation [15,16,17,18], transcriptional factor regulation [19,20], epigenetic regulation [21,22], and signal transduction regulation [23]. They can effectively regulate *Fusarium* secondary metabolite production. These fungal species, mainly including *F. avenaceum* [24], *F. fujikuroi* (teleomorph: *Gibberella fujikuroi*) [25], *F. graminearum* (teleomorph: *G. zeae*) [26,27], *F. oxysporum* [28,29,30], *F. proliferatum* (teleomorph: *G. intermedia*) [31,32], *F. pseudograminearum* [33], *F. sulphureum* [34], *F. verticillioides* (teleomorph: *G. moniliformis*) [31,35], have been well studied for their secondary metabolite production via biosynthesis regulation.

In the past three decades, many advances have been achieved in the regulation on secondary metabolite production with the continuous revelation of biosynthetic gene clusters (BGCs) related to secondary metabolism in *Fusarium* fungi. To our knowledge, the specific reviews about the strategies for secondary metabolite production via biosynthesis regulation on *Fusarium* fungi have not been reported. This review summarizes diverse strategies, including regulations of environmental factors, global and pathway-specific transcriptional factors, epigenetic modification, signal transduction, the use of organic chemicals, and plant/microorganism-derived extracts to modulate secondary metabolite production in at least 50 *Fusarium* species. These approaches aim to discover novel bioactive secondary metabolites, inhibit harmful mycotoxins, and expedite the practical application of valuable metabolites derived from *Fusarium* fungi.

## 2. Regulation of Environmental Factors on *Fusarium* SM Production

The environmental factors can activate or suppress fungal BGCs to either increase or decrease production of SMs for fungal physiological or ecological adaptation to the environment. These environmental factors mainly include light, temperature, water availability (activity), ambient pH, carbon and nitrogen sources, and other media components [36,37]. Sometimes, they are known as the OSMAC (one strain many compounds) approach that the fungal cryptic metabolite BGCs are activated. OSMAC means the modulation of the optimal culture conditions (i.e., media composition, carbon and nitrogen sources, light, temperature, pH, and osmolarity) for SM production in fungi [38]. In addition, the transcriptional, signal transduction and epigenetic regulations of the genes involved in the biosynthetic pathways of SMs in fungi are responsive to environmental stimuli. Therefore, the environmental factors can affect the production of fungal SMs including their composition and relative contents. Some fungal species such as *F. fujikuroi* [25,39,40], *F. graminearum* [41], *F. proliferatum* [31], *F. temperatum* [42] and *F. verticillioides* [31,43] have been well studied for the environmental factors that affect their SM production. In fact, various environmental factors synergistically regulate the biosynthesis of SMs in fungi. Here, we summarize the effects of environmental factors on fungal secondary metabolism mainly based on the similar regulation mechanisms that a certain type of environmental factor may have.

### 2.1. Regulation by Light

By sensing and regulating gene expression modulated through light, fungi can produce various bioactive metabolites [16,17,18,44,45]. The impacts of light regulation on the production of some *Fusarium* SMs, such as carotenoids and gibberellins, have been reviewed previously [46].

Light repressed fusarin production via white collar protein WcoA in *F. fujikuroi* [47]. Further study showed that WcoA and WcoB regulated the mRNA levels of various genes in *F. fujikuroi*, including the genes for the photoreceptors OpsA and CryD, the regulatory proteins Csp1 and Hog1, and some key enzymes in the biosynthesis of different SMs, such as beauvericin (BEA), carotenoids, and fusarins. However, the deletion of *WcoA* and *WcoB* genes had little effect on the key biosynthetic genes of bikaverin and gibberellins in *F. fujikuroi* [48].

*F. fujikuroi* contained a gene encoding a speculated cry-DASH, namely CryD. The expression of the gene *cryD* in the wild-type (WT) strain was induced by light. The WT strain exhibited moderate photo-induction of gibberellin production, while the Δ*cryD* mutant did not [49]. VvdA was involved in the light regulation of fungal development and affected the accumulation of carotenoids in *F. fujikuroi*. The absence of *vvdA* in *F. fujikuroi* resulted in a shallow pigmentation under constant light exposure. The targeted Δ*vvdA* mutants accumulated fewer carotenoids than the WT strain [50]. In addition, *F. fujikuroi* could produce carotenoids which were terpenoid pigments acting as antioxidants and photoprotectants. The biosynthesis of carotenoids was stimulated by light via the regulation of the gene *carS*, which demonstrated that the transcription of the gene *carS* was positively regulated by light [51].

Both *Fgwc-1* and *Fgwc-2* genes were essential for light-dependent processes of *F. graminearum*. If these two genes were deleted, the biosynthesis of aurofusarin and trichothecene was derepressed [52].

The photolyase gene *phr1* from the phytopathogen *F. oxysporum* f. sp. *lycopersici* was induced by visible light. Both the expression of the gene *phr1* and the presence of α-tomatine were detected in the fungus. α-Tomatine was a glycoalkaloid from tomato damaged cell membranes, indicating that *phr1* was induced by this cellular stress [53].

Red and green lights favored the production of antimicrobial compounds 8-deoxyjavanicin and fusolanone A in *F. solani*, an endolichenic fungus. It indicated that the adaptability of fungi to light offered an alternative in using light as an effective and low-cost approach to regulate and induce biosynthesis of beneficial bioactive compounds in fungi [54].

### 2.2. Regulation by Temperature

Temperature significantly affected the growth and metabolism of *Fusarium* fungi. The optimal temperature for the production of fumonisin was between 15 °C and 25 °C for *F. proliferatum*, and between 20 °C and 30 °C for *F. verticillioides* [55,56]. The expression of the fumonisin biosynthetic *FUM1* gene was markedly induced at 20 °C in both *F. proliferatum* and *F. verticillioides*. However, the optimal temperature for fungal growth was 25 °C for these two *Fusarium* species [57].

*F. subglutinans*, which was isolated from maize ear rot materials in Poland, was cultured at different temperatures on a few cereal substrates (barley, maize, wheat, rye, oat, and rice kernels) as media. Among these substrates, rye substrate favored fusaproliferin production, wheat and rice substrates favored BEA production, and rice substrate favored moniliformin production. When *F. subglutinans* was cultured on rice substrate, the fungus produced the highest levels of BEA and fusaproliferin at 20–25 °C, while moniliformin production was the most suitable at 30 °C [58].

*Fusarium* species had the maximal production of fumonisins B1 (FB1) and B2 (FB2) at 20–25 °C on Czapek yeast agar plus 5% salt or potato dextrose agar (PDA) [59]. Similar results were observed for *F. verticillioides* to produce FB1 and FB2 at 20–35 °C [60]. Another study showed that the suitable conditions for *F. verticillioides* growth were 25 °C with water availability as 0.98 a_w_, whereas the highest FB1 yield was observed at 15 °C with water availability as 0.98 a_w_ [61].

### 2.3. Regulation by Water Availability

Regulation of water availability/activity (a_w_) on the expression of biosynthesis genes of *Fusarium* SMs has also been studied. Generally, temperature and water availability (relative humidity) synergistically affected fungal growth and SM accumulation. The optimal water availability for fumonisin production was 0.97–0.98 a_w_ for *F. proliferatum* and *F. verticillioides* [55,56].

The influences of different water availability and temperature on the contents of free and conjugated zearalenone (ZEA or ZEN) and deoxynivalenol (DON) for the stored wheat inoculated with *F. graminearum* were studied. As an important conjugate of DON, there was a significant difference in the content of DON-3-glucoside and its precursor DON in naturally contaminated wheat at 0.93 a_w_ and 25 °C, with a ratio of 56:44, respectively. The high contents of DON-3-glucoside could be influenced by the wheat varieties, harvesting seasons, fungal strain types, and locations. Unexpectedly, the content of ZEN-14-sulfate was three times higher than that of ZEN in naturally contaminated wheat at 0.98 a_w_. The contents of emerging mycotoxins such as moniliformin were increased with increasing temperature and reached their highest levels at 0.95 a_w_ and 25 °C. In general, water availability had a significant impact on the content of each mycotoxin, while temperature changes had no significant effect on the content of each mycotoxin [62].

The effects of water availability (a_w_), temperature, incubation time and their interactions on the accumulation of mycotoxins as well as the expression levels of biosynthetic genes in *F. graminearum* species complex strains from maize samples were studied. At 0.98 a_w_/30 °C or 0.99 a_w_/25 °C, the highest contents of DON, 3-AcDON and 15-AcDON of the *F. boothii* and *F. graminearum* strains were observed. The maximum contents of nivalenol (NIV) and 4-AcNIV were achieved at 0.99 a_w_ and 30 °C in *F. asiaticum* and *F. meridionale* [63]. Another example was the effect of temperature and water availability on mycotoxin production by *F. oxysporum* and *F. sambucinum* responsible for dry rot in potato tubers. The mycotoxins, including T-2, HT-2, diacetoxyscirpenol (DAS), 15-acetoxyscirpenol (15-AS), neosolaniol, and BEA were easily examined when potato tubers were stored at 10 °C and 0.99 a_w_ for 21 days. The relative contents of mycotoxins of the potato tubers at 0.99 a_w_/10 °C were much higher than those of the potato tubers at 0.97 a_w_/5 °C [64].

The highest level of fusaric acid (FA) was detected at 0.995 a_w_/25 °C in grain contaminated with *F. temperatum*. Drying grain was the best strategy to reduce FA and fusarinolic acid contamination of grains, as the fungal growth and mycotoxin accumulation were typically at low levels [42].

The effects of water availability (0.955 a_w_ and 0.990 a_w_) on the expression of five genes (i.e., *FUM3*, *FUM8*, *FUM13*, *FUM14* and *BIK1*) in *F. verticillioides* were investigated after 14 and 21 days of cultivation, respectively. The FB production and biosynthetic gene expression reached their maximum values at 0.990 a_w_, and the bikaverin production and *BIK1* expression also showed the same trend. *FUM3* and *FUM14* were the most highly expressed genes, positively correlated with the production of FB1, FB2, and FB3 [65].

### 2.4. Regulation by Ambient pH

Ambient pH is an important environmental factor affecting the growth and development of *Fusarium* fungi, and their SM production. In *F. fujikuroi*, acidic environments (pH 4–5) promoted production of fusaric acid (FA), a virulence factor that disrupted plant membrane integrity [66,67]. Conversely, alkaline conditions suppressed melanin synthesis, reducing fungal survival under UV stress in *F. fujikuroi* [68].

In the liquid culture of *F. proliferatum*, the optimal pH range for fumonisin production was from 3.0 to 3.5. However, when pH was higher than 3.5, the growth of fungi would be enhanced [69].

Both *Tri* (*TRI*) gene expression and trichothecene B biosynthesis were induced by acidic pH (i.e., pH 3, 4 and 5) in *F. graminearum*, the pathogen of wheat *Fusarium* head blight and maize *Fusarium* ear rot. When the expression of the *FgPAC1* gene, a zinc finger transcription factor, was high, the expression of *Tri* genes was repressed [70,71].

The ambient pH 6 was beneficial for the growth, pathogenicity, and diacetoxyscirpenol (DAS) production of *F. sulphureum*, which was the pathogen to cause *Fusarium* rot of muskmelon. Ambient pH 6 was also more conducive to the secretion of cell wall-degrading enzymes of the pathogen to degrade the cell wall of the host plant and upregulated the expression of DAS biosynthesis genes [34].

### 2.5. Regulation by Carbon Sources

Seven monosaccharides (i.e., fructose, arabinose, galactose, xylose, sorbose, mannose, and glucose), five disaccharides (i.e., sucrose, cellobiose, trehalose, maltose, and lactose), and three polysaccharides (i.e., dextrin, xylan, and inulin) were used as the carbon sources in the media to test their influences on secondary metabolism in three *F. avenaceum* strains. The fungal strains could grow and produce aurofusarin on the tested carbon sources. Enniatins (ENs) and moniliformin were produced on all carbon sources except on lactose, which indicated a common conserved regulatory mechanism. Differences in the production of fusarin C, chlamydosporol, 2-AOD-3-ol, and antibiotic Y were observed among *Fusarium* strains, indicating that the carbon source played a regulatory role in their biosynthesis [72].

Glucose promoted the growth of *F. fujikuroi* by shutting down SMs. Thus, the production of gibberellins was affected by the addition of glucose as the sole carbon source. However, the mixture of carbon sources could promote the slow assimilation of gibberellic acid (GA3) but increase its yield, demonstrating the influence exerted by the carbon metabolism [73].

Mannose, used as the sole carbon source, significantly blocked the production of FB1 and FB2 by *F. proliferatum* as compared with the addition of sucrose. The RT-qPCR analysis indicated that expression of several key genes involved in the FB biosynthetic pathway and in transcription regulation was significantly downregulated in *F. proliferatum* with mannose as the carbon source, whereas the expression of histone deacetylation-related genes was significantly upregulated. These results indicated that the blockade of FB biosynthesis by mannose was related to the reduced conversion of acetyl-CoA to polyketide biosynthesis [74,75]. Further investigation showed that *F. proliferatum* cultivated in the Czapek’s broth (CB) without sucrose greatly induced production of fumonisins, while additional sucrose supplementation in the medium significantly decreased the production of fumonisin. In addition, cellulose, hemicellulose, and other polysaccharides extracted from banana peels replaced sucrose as a carbon source, reducing the production of fumonisins by *F. proliferatum*. Correspondingly, the genes related to fumonisin synthesis, such as *FUM1* and *FUM8*, were significantly upregulated in the culture medium without sucrose [76]. In contrast, compared with sucrose, glucose had no significant effect on the fungal growth and fumonisin production of *F. proliferatum*, indicating that the glucose-responsive repressor CreA might not be a key regulatory factor in fumonisin biosynthesis [74].

It was reported that the starch content in maize affected the production of FB1, and the deletion of the α-amylase gene, *AMY1*, in *F. verticillioides* resulted in a decrease in FB1 production in starchy kernels [77]. Another investigation showed that among five carbon sources (i.e., glucose, amylopectin, amylose, starch, and amylose), the maximum production of FB1 was achieved with glucose, while the maximum production of sesquiterpenes was achieved with amylopectin in *F. verticillioides* [78].

*F. verticillioides* was a producer of useful SMs such as naphthoquinone pigments, monoterpenes, and sesquiterpenes. Their biosyntheses were stimulated in the cultures with the addition of fructose, lactose, and xylose at their optimal concentrations, respectively, with fumonisins being absent or present in trace amounts. However, the highest biosynthesis of fumonisins occurred in the medium with the addition of sucrose. The concentrations of FB1 and FB2 reached 7.85 mg/g dw and 0.38 mg/g dw, respectively [79].

It was concluded that carbon sources significantly affected SM production in *Fusarium* fungi. In general, fungi grew better with glucose than with other carbon sources. For the SM production in *Fusarium* fungi, the carbon catabolite repression (CCR) should be a major influence on the carbon-source-mediated regulation of metabolite biosynthesis [78,79].

### 2.6. Regulation by Nitrogen Sources

In fungi, different sources of nitrogen supplements resulted in physiological, morphological and metabolic alterations [15]. Some *Fusarium* fungi can produce naphthoquinone pigments such as fusarubin and bikaverin. The production of fusarubin was favoured in nitrogen -limited conditions by *F. chlamydosporum* [80].

The production of some PKS-NRPS-derived mycotoxins (i.e., fusarin C) in *F. fujikuroi* was usually regulated under high-nitrogen and acidic pH conditions [81]. The production of GA3 was repressed by the presence of nitrogen in high amounts of glutamine in *F. fujikuroi* [82]. Nitrogen starvation increased carotenoid accumulation in wild-type (WT) and carotenoid-overproducing strains [83].

With some amine compounds such as arginine, ornithine, putrescine and agmatine added in the media for cultivation of *F. graminearum*, the DON concentrations in the media were extremely high, and were equal to, or greater than 1000 mg/L [84]. Further mechanism study showed that two of the most strongly *Tri6*-dependent and agmatine-co-regulated genes appear to negatively regulate DON production [85]. When the medium pH was maintained at 4.0 in *F. graminearum*, and three amino acids including glycine, serine and threonine were added to the medium, respectively, they all suppressed the production of trichothecenes [86].

The production of phytohormone cytokinin in *F. pseudograminearum* was enhanced by PM3 as the nitrogen source. DON production was also increased in both *F. graminearum* and *F. pseudograminearum* by specific nitrogen sources [87].

The UHPLC-MS/MS analysis indicated that nitrogen sources (i.e., urea, NaNO_3_, and (NH_4_)_2_SO_4_) affected gibberellin biosynthesis and metabolic flux in *F. sacchari*. Additionally, the transcriptome analysis elucidated the potential impact of nitrogen availability on the expression of several genes involved in the synthesis of *F. sacchari* mycotoxins [88].

### 2.7. Regulation by Other Environmental Factors

Other environmental factors such as oxidative stresses [89], osmotic stresses [90], metal ions [91], medium components [43,92], and their complex factors, can regulate fungal SM production as well.

#### 2.7.1. Regulation by Oxidative Stresses

When the liquid cultures of *F. graminearum* were treated with H_2_O_2_, the accumulation of trichothecenes including DON and 15-AcDON was rapidly and strongly enhanced. Due to H_2_O_2_ being the main factor causing oxidative bursts in pathogen-host interactions, this supported the theory that trichothecenes served as the virulence factors in the pathogenetic process [89].

#### 2.7.2. Regulation by Osmotic Stresses

The production of trichothecenes in *F. graminearum* was markedly inhibited by NaCl which had no obvious effect on fungal growth. Both the osmosensor histidine kinase and osmotic stress-activated protein kinases were found to positively regulate aurofusarin production and negatively regulate trichothecene production [90].

#### 2.7.3. Regulation by Metal Ions

Some metal ions have been revealed to affect SM production in *Fusarium* fungi. Mg^2+^ and Mn^2+^ suppressed trichothecene production at relatively low concentrations, while Fe^2+^, Zn^2+^ and Co^2+^ enhanced trichothecene production at relatively high concentrations in *F. graminearum* [91,93,94]. Mechanism study showed that Co^2+^ stimulated production of trichothecene by activating *Tri6* transcription in *F. graminearum* [91].

Iron starvation induced biosynthesis of ferrichrome in *F. oxysporum* f. sp. *cubense* (*Foc*) TR4. It indicated that *Foc* TR4 produced hydroxamate, siderophore, and ferrichrome in response to iron starvation [95].

#### 2.7.4. Regulation by Other Additives

Four PDA media from different manufacturers such as VWR, Fluka, Sigma, and Oxoid were used to examine their effects on the metabolite profiles of four *Fusarium* species (i.e., *F. pseudograminearum*, *F. graminearum*, *F. fujikuroi*, and *F. avenaceum*) using HPLC-HRMS analysis, from which the significant differences in intensity of nine out of ten metabolites were observed [92].

The poor nitrogen sources, alkaline pH, low iron availability, and CWI MAPK signaling were proven to be associated with increased production of fusaric acid in *F. oxysporum* [96].

Fusarielin-type polyketides are a therapeutically promising class of *Fusarium* metabolites. The combination of disaccharides, dextrin and arginine significantly increased the yield of fusarielin in *F. graminearum* and *F. tricinctum* [97].

The endophytic fungus *F. tricinctum* was cultured on the solid rice medium supplemented with fruit and vegetable juices, which resulted in an 80-fold increase in the accumulation of fusarielins A, B, J, K and L. However, when the fungus was grown in rice media lacking vegetable juice or fruit juice, fusarielin J was screened. In the presence of apple juice and carrot juice, the accumulation of fusarielin J was observed to be the most, while the stimulating effect of banana juice was weaker [98]. A similar study found that fermentation of *F. tricinctum* in solid beans and liquid Wickerham medium versus cultivation on solid rice medium resulted in an increase in the production of enniatins in the cultures of *F. tricinctum* with solid beans [99].

The influences of the substrates on mycotoxin production by *F. verticillioides* were studied. Maize meal agar medium was beneficial to the production of fumonisins A1 and B1, while malt extract agar was conducive to the production of fumonisins A2 and B2. Other mycotoxins such as fusarins, bikaverin derivatives and fumonisin analogs with different growth conditions were also identified [43].

## 3. Global and Pathway-Specific Transcriptional Factor Regulation on *Fusarium* SM Production

In the biosynthesis of fungal SMs, transcriptional factors (TFs) are generally divided into two groups. The first group is global TFs, which are basically located outside SM BGCs, controlling a variety of secondary metabolic pathways. These TFs mediate fungal responses to environmental signals. Another group are pathway-specific TFs, which are basically located in the specific SM BGCs and affect gene expression in the clusters. In *Fusarium* fungi, the global TFs are in response to carbon and nitrogen sources, ambient light, and pH [100], the pathway-specific TFs mainly regulate the expression of secondary metabolism-related genes in the cluster where they are located [101].

### 3.1. Global Transcriptional Factor Regulation

The global TFs are able to regulate secondary metabolism in addition to regulating mycelial differentiation, sporulation, and other developments. The global TFs can not only sense the changes of nutrient components in the media, but also the changes of environmental signals. They are capable of associating with other TFs for transmission, finally regulating multiple secondary metabolic BGCs in fungi to adapt to environmental changes [100]. Some global TFs have been reported to regulate secondary metabolism including LaeA, LaeB, velvet proteins, AreA, AreB, and PacC in *Fusarium* fungi.

#### 3.1.1. Regulation of LaeA, LaeB and Velvet Proteins

Global transcriptional regulators in response to ambient light in *Fusarium* fungi included LaeA, LaeB and velvet proteins (i.e., VeA, VelB and VelC). Among these transcriptional factors, VeA was considered the central player of the light regulatory network in *Fusarium* species [18].

1. Regulation of LaeA and LaeB

*F. fujikuroi* was the pathogen of rice bakanae to produce many SMs such as bikaverin, gibberellins, fusaric acid, fusarubins, and fusarins. Among them, fusaric acid and fusarins belonged to the mycotoxins, and gibberellins belonged to phytohormones [66]. LaeA had a positive regulatory effect on the production of certain metabolites in *F. fujikuroi*. For example, deletion of the *FflaeA* gene in *F. fujikuroi* resulted in a decrease in the production of gibberellins A3 and A4, fusarin C, fumonisins B1, B2, B3 and B4, DON, and 15-AcDON [102]. Subsequently, similar results have been confirmed. Deletion of the *Fflae1* (*FflaeA*) gene led to a reduction in the production of fusaric acid, fusarinolic acid, and dehydrofusaric acid in *F. fujikuroi* [103]. In addition, the deletion of *Fflae1* led to a decrease in the production of gibberellins, fumonisins and fusarin C. Overexpression of *Fflae1* resulted in an increase in the production of gibberellins in another *F. fujikuroi* strain [104]. Sometimes, LaeA negatively regulated some metabolite production in *F. fujikuroi*. The deletion mutant Δ*FflaeA* of *F. fujikuroi* showed an increase in the yield of bikaverin [102]. Another example was that deletion of the *lae1* gene in *F. fujikuroi* led to upregulation of gibepyrone BGC expression as well as increased production of gibepyrones A, B, C, D, E, and F [105].

LaeA positively regulated the production of metabolites in the following plant pathogenic *Fusarium* species. Deletion of *FglaeA* in *F. graminearum* led to a dramatic decrease in the production of trichothecenes and zearalenone. Overexpression of *FglaeA* caused an increase in the production of trichothecenes and zearalenone, which indicated that FgLaeA positively regulated production of phytotoxins by *F. graminearum* [28]. For *F. oxysporum* f. sp. *niveum*, the deletion of the *FoLae1* gene led to a decrease in conidia yield, as well as a reduction in the production of bikaverin and fusaric acid. In addition, all these changes in the deleted mutants were restored in the corresponding complementation strains [29]. For *F. oxysporum*, the deletion of the gene *laeA* led to a decrease in the production of BEA and fusaric acid (FA), which contributed to the virulence to plant hosts such as tomato plants [106]. For *F. verticillioides*, the deletion of the *laeA* gene reduced production of fusarin C, bikaverin, fusaric acid and fumonisins [107].

LaeB, an orthologue similar to LaeA, was first identified using a forward genetic screening in *Aspergillus nidulans* [108]. LaeB was involved in regulating the production of sterigmatocystin and other polyketides [109]. FpLaeB, an orthologue of LaeB protein, was required to regulate the secondary metabolism in *F. pseudograminearum*. The generation of DON was impaired in the *FpLaeB* deletion mutant via UHPLC-MS/MS assay. FpLaeB was also important for the formation of conidia as the *FpLaeB* deletion mutant formed fewer conidia in the induced medium. In addition, the *FpLaeB* deletion mutant showed reduced sensitivity to the cell wall integrity inhibitors, and its growth was more severely inhibited by the cell membrane inhibitor sodium dodecyl sulfate (SDS) than that of the wild-type strain. More importantly, when the Δ*FpLaeB* mutant was inoculated on the stem base or head of wheat, its virulence was decreased. These results indicated that FpLaeB played an important role in the growth, development, and maintenance of the cell walls, as well as membrane integrity. More importantly, FpLaeB was necessary for SM production and complete virulence of *F. pseudograminearum* [110]. Some examples of LaeA and LaeB modulating SM production in *Fusarium* fungi are shown in Table 1.

2. Regulation of Velvet Proteins VeA, VelB and VelC

The deletion of the *veA* gene in *F. fujikuroi* resulted in a decrease in the production of fusarin C, fumonisins B1, B2, B3 and B4, DON, 15-AcDON, and gibberellins A3 and A4. However, in the *veA* deletion strain, there was an increase in the production of bikaverin [102]. The subsequent results showed that the deletion of *Ffvel1* (*FfveA*) resulted in a decrease in the production of fusarinolic acid, fusaric acid, and dehydrofusaric acid in *F. fujikuroi* strain [103]. Deletion of *vel1* resulted in a decrease in the production of gibberellins, fumonisins and fusarin C. Overexpression of *lae1* increased in the production of gibberellins in another *F. fujikuroi* strain [104]. The deletion of *vel1* gene in *F. fujikuroi* led to the upregulation of gibepyrone BGC expression, and an increase in the production of gibepyrones A, B, C, D, E, and F as well [105].

Deletion of *veA* in *F. graminearium* resulted in a decrease in the production of DON (also called vomitoxin) [111], and also led to a decrease in the production of trichothecenes [112].

The overexpression of *FnveA* in *F. nematophilum* led to an increase of the antitumor activity of the crude extract against A549 cancer cells. Unfortunately, the antitumor compounds needed further identification [113].

The deletion of *veA* in *F. oxysporum* led to a decrease in the production of fusaric acid and beauvericin, which resulted in virulence to plant hosts such as tomato plants [106]. The deletion of the *Fovel1* gene in *F. oxysporum* f. sp. *niveum* resulted in a decrease in the number of conidia, and a decrease in the production of fusaric acid and bikaverin. Furthermore, all these alterations in the deleted strains were restored in the corresponding complementary strains [29].

The global regulator VeA in *F. solani*, an endophytic fungus isolated from the medicinal plant *Nothapodytes pittosporoides* (Icacinaceae), was overexpressed. The antitumor activity of the crude extract was greatly increased. Metabolomics analysis showed that there were 48 key genes related to antitumor activity. Unfortunately, the antitumor compounds were not identified in the extract [114]. Another global regulator VeA was found to negatively regulate the transcription factor MtfA, which in turn targeted negatively regulating transcriptional levels of PRPS2 to mediate acadesine (AICAR) biosynthesis in *F. solani* [115].

When the *Fvvel1* (*FvveA*) gene was deleted in *F. verticillioides*, the production of gibberellins, fusarin C, fumonisins B1, B2, B3 and B4, DON, and 15-AcDON was decreased. However, in the Δ*Fvvel1* mutant, the production of bikaverin was increased. The mechanisms of the gene *Fvvel1* on the aforementioned metabolite production should be similar to those of *FvlaeA* in *F. verticillioides* [102]. The deletion of *veA* in maize pathogen *F. verticillioides* led to a decrease in the production of fusarin C, fumonisins B1, B2 and B3 [116]. Further investigation indicated that VeA was necessary for causing symptoms and mycotoxin synthesis in maize seedlings by *F. verticillioides* [117].

When the *vel2* (*velB*) gene was deleted in *F. fujikuroi*, gibepyrone BGC expression was upregulated, and the production of gibepyrones A, B, C, D, E, and F was also increased in *F. fujikuroi* [105].

When the gene *FgvelB* was deleted in *F. graminearum*, the production of DON was decreased [118]. Production of trichothecenes and ZEN in the Δ*FgvelB* mutant of *F. graminearum* was also significantly reduced compared with the WT strain [119]. A similar example was that the deletion of *FpvelB* led to singificant differences in growth, conidiation, virulence and production of DON in *F. pseudograminearum*. In addition, FpVelB positively regulated another SM BGC associated with pathogenesis by modulating the expression of the gene *PKS11*. FpVelB regulated pathogen virulence by influencing DON biosynthesis in *F. pseudograminearum* [33].

*F. proliferatum* was the causative agent of rice spikelet rot disease. The disruption of *FpvelC* enhanced the production of fumonisin B1 and fusaric acid concomitantly. The transcripts of the BGC genes responsible for the biosynthesis of two mycotoxins were also significantly increased [120]. Some examples of VeA, VelB and VelC regulating SM production in *Fusarium* fungi are shown in Table 2.

#### 3.1.2. Regulation of AreA and AreB

AreA and AreB are global regulators belonging to the GATA transcriptional factor family in response to nitrogen sources that can modulate the production of SMs in fungi [121].

AreA could activate GA3 biosynthetic genes under nitrogen limitation in *F. fujikuroi* [122]. Both AreA and AreB were GATA-type transcriptional factors, which were studied in detail in *F. fujikuroi*. *FfAreA* shared 98% homology with the Cys_2_/Cys_2_ zinc finger domain of homologous fungi and acted as a GATA TF, directly binding to GATA/TATC elements in the promoter regions of six biosynthetic genes in the GA biosynthetic gene cluster. Therefore, AreA was considered the main regulator of GA3 biosynthesis, explaining the relationship between dynamic nitrogen status and yield [123]. By isolating, characterizing, and destroying AreA from *F. fujikuroi*, the dominant role of AreA in primary and secondary metabolic regulation was further revealed [124]. The transcriptional level and subcellular localization of AreA were preliminarily determined by the extracellular/intracellular nitrogen status. The accumulation of AreA in nuclear localization could continuously stimulate nitrogen metabolism, serving as a transcriptional factor targeting nitrogen-availability related genes [123].

AreB was identified as a negative regulatory factor of AreA-dependent genes in *F. fujikuroi* [125]. AreB acted as both repressor and activator of AreA-dependent genes, and three transcripts, including *FfareB-a*, *FfareB-b*, and *FfareB-c*, were discovered through alternative splicing with differential expression levels [126]. Further exploration was conducted on the subcellular localization of three transcripts and their interaction patterns with AreA. In most cases, the localization patterns of AreB-b and AreB-c were similar to AreA, while the localization of AreB-a under nitrogen-inhibited growth conditions was different. Fluorescence microscopy of AreB subcellular localization showed that AreB-a was the only nuclear localization. On the contrary, only a few *F. fujikuroi* hyphae showed nuclear localization fluorescence signals of AreB-b and AreB-c [126]. The nuclear localization heterodimers of AreA and AreB bound to the GATA/TATC elements of the target gene promoter and upregulate the biosynthesis of GA3 under nitrogen deprivation conditions [126]. The deletion of *areA* in *F. fujikuroi* revealed that nearly 24.5% of annotated transcription factors (TFs) were affected by nitrogen starvation, and 30% of TFs were affected by *areB*- deficiency during nitrogen starvation or nitrogen sufficiency [127].

AreA exhibited critical roles in regulating the production of DON by ammonium and cyclic adenosine monophosphate (cAMP) signaling in *F. graminearum*. The vegetative growth and DON yield of the Δ*areA* mutant were significantly reduced in *F. graminearum* cultures. The interaction between AreA and Tri10 (TRI10) might be related to its role in regulating *Tri* gene expression. Further research suggested that AreA participated in regulating DON production through ammonium inhibition and the cAMP-PKA pathway [128].

Another study showed that the Δ*FgareA* mutation triggered loss of trichothecene biosynthesis but did not affect zearalenone biosynthesis in *F. graminearum* [129]. Furthermore, overexpression of area increased production of gibberellins and bikaverin in *F. graminearum* [130].

AreA contributed to chromatin accessibility and expression of two velvet-regulated BGCs, encoding the biosynthesis of BEA and ferricrocin in *F. oxysporum* [131].

*F. proliferatum* was the pathogen of rice spikelet disease. The Δ*FpareA* mutant of *F. proliferatum* did not utilize nitrate as the N source, but instead utilized ammonium (NH_4_^+^) or glutamine as the N source. Except for using 120 mmol/L of ammonium chloride (NH_4_Cl) as the N source, the ability of the Δ*areA* mutant to biosynthesize fumonisin was significantly reduced [132].

The Δ*areA* mutant could not produce FB1 under either low or high nitrogen levels in *F. verticillioides*, which indicated that AreA profoundly affected fumonisin biosynthesis [133]. The deletion of the gene *FUG1* reduced the production of fumonisins (i.e., FB1, FB2, and FB3) in *F. verticillioides*. Further RNA-seq analysis showed that AreA was downregulated in the *FUG1* deletion strain of *F. verticillioides*, indicating that *FUG1* might affect fumonisin biosynthesis by directly or indirectly regulating AreA. These results collectively provided important evidence that AreA and/or nitrogen sources regulated fumonisin biosynthesis [134]. Some examples of AreA and AreB regulating SM production in *Fusarium* fungi are shown in Table 3.

#### 3.1.3. Regulation of PacC

PacC belonged to the Cys_2_His_2_ zinc finger family and recognized the DNA sequence 5-GCCARG-3. It was a key TF for fungal pH regulators [135]. PacC could regulate the production of various SMs in *Fusarium* fungi. Deletion of the pH regulatory gene *pacC* in *F. fujikuroi* resulted in partial derepression of the *bik* gene at acidic ambient pH, and led to a significant reduction in bikaverin synthesis [136].

The production of trichothecene was induced only under acidic pH conditions in *F. graminearum*. The Δ*FgPac1* mutant was constructed, which showed decreased development at neutral and alkaline pH, increased sensitivity to H_2_O_2_ and an earlier induction of *Tri* gene and toxin accumulation at acidic pH. The strain expressing the *FgPac1c* constitutively active form of Pac1 exhibited strongly repressed *Tri* gene expression and reduced mycotoxin accumulation at acidic pH. The results demonstrated that Pac1 negatively regulated *Tri* gene expression and mycotoxin production in *F. graminearum* [137].

The pH of the environment surrounding *F. proliferatum* cells could affect the production of FB1 and FB2 as well as the expression of *FUM*. Further investigation on the molecular mechanism of fumonisin synthesis in *F. proliferatum* indicated that different pH conditions led to the production of different fumonisins. It was also noted that some changes in protein accumulation were paralleled by the production pattern of fumonisins. Further analysis of the potential functions of these proteins suggested that they might be related to SM biosynthesis and the structural modifications of fumonisins. Therefore, these differential responses indicated that the biosynthesis of fumonisin played a mediating role under different pH conditions [138].

Deletion of *PAC1* in *F. verticillioides* induced an increase in the production of FB1 and in the expression of *FUM1* when the fungus was cultured on maize kernels under acidic pH conditions, indicating the regulatory role of *PAC1* in FB1 biosynthesis [139].

### 3.2. Pathway-Specific Transcriptional Factor Regulation

According to the genome information obtained, about 60% of fungal secondary metabolism BGCs contain pathway-specific transcriptional factors (TFs) that regulate the expression of secondary metabolism-related genes in the cluster. 90% of pathway-specific TFs belong to zinc finger proteins [140].

Overexpression of the pathway-specific TF gene *FvFum21* in *F. fujikuroi* strongly activated the *FUM* cluster genes, leading to a 1000-fold increase in FBx levels [141].

Two pathway-specific Zn(II)_2_Cys_6_-type TFs, namely Fub10 and Fub12, were involved in fusaric acid BGC in *F. fujikuroi*. Fub10 positively regulated the expression of all *FUB* genes, while Fub12 participated in the bioconversion of the two fusaric acid derivatives, i.e., dehydrofusaric acid and fusarinolic acid, as a biotransformation detoxification [142].

GIP2 was a pathway-specific TF that regulated the aurofusarin BGC in *F. graminearum*. The analysis of targeted gene deletion and complementation confirmed that *GIP2* was needed for the biosynthesis of aurofusarin. Overexpression of *GIP2* in the wild-type strains increased aurofusarin production and reduced mycelial growth [143].

Activation of the local transcription factor FSL of the polyketide synthase 9 (PKS9) cluster led to production of fusarielins F, G and H in *F. graminearum*. The cytotoxicity of the three fusarielins was studied against colorectal cancer cell lines. Among them, fusarielin H showed more cytotoxic activity than fusarielins F and G [144].

Trichothecenes are isoprenoid mycotoxins isolated from wheat materials infected with the *F. graminearum*. Deletion of two pathway-specific TFs *Tri6* and *Tri10* led to greatly reduced production of toxins [71,145]. The expression of both *Tri6* and *Tri10* genes was later found to be stimulated by cyclic adenosine monophosphate (cAMP) treatment, which indicated that *Tri6* and *Tri10* genes were crucial for the regulation of DON biosynthesis by cAMP signaling in *F. graminearum* [146]. Tri6 (TRI6) was previously considered a global TF in *F. graminearum* [147,148], and later was recognized as a pathway-specific TF [100]. Tri6, a pathway-specific Zn(II)_2_Cys_6_-type TF in *F. graminearum*, directly bound to trichothecene BGC promoters under host-mimicking conditions, coordinating DON synthesis with infection stages [41,149].

ZEB2 was a pathway-specific TF belonging to the bZIP family. *ZEB2* expression positively regulated zearalenone production in *F. graminearum* [150].

The *FUM* cluster gene *fum21* encoded a Zn(II)_2_Cys_6_-type TF. The production of fumonisins was decreased in the knockout mutant Δ*Fvfum21* of *F. verticillioides* [151]. Some examples of the pathway-specific transcriptional regulation of *Fusarium* secondary metabolism are shown in Table 4.

### 3.3. Miscellaneous Transcriptional Factor Regulation

Most eukaryotic transcriptional factors (TFs) could be classified into different groups based on the types of DNA-binding domains. They included basic region/leucine zipper (bZIP), MADS box, myb, homeobox helix–loop–helix, and zinc fingers. Among them, bZIP TFs were involved in fungal stress response, asexual development and other cell processes [152]. Most of the transcriptional factor regulations belong to the global regulations in response to environmental stresses. Other reported miscellaneous global regulations in *Fusarium* species include regulations of Sge1, HXK1, and AtfA.

The *esyn1* gene was responsible for the modulation of enniatins (ENs) biosynthesis in *F. avenaceum*. Activation of *esyn1* transcription led to increased production of ENs [24].

MeaB belonging to the bZIP TF demonstrated a completely different regulatory capability from Cpc1 [153]. Two distinct MeaB TFs, MeaBL and MeaBS from *F. fujikuroi*, were expressed in an AreA-dependent manner, which depended on the availability of nitrogen. During the nitrogen-sufficient period, especially with the addition of glutamine, MeaBL appeared more frequently in the nucleus, while MeaBS appeared to be dysfunctional as it remained isolated in the cytoplasm [154]. Under nitrogen starvation, blocking MeaB could slightly upregulate GA cluster genes and some intracellular nitrogen transport channels [153].

In *F. fujikuroi*, the global regulator FfSge1 was required for expression of SM gene clusters, but not for conidiogenesis and pathogenicity. Its overexpression in the wild-type background led to increased production of fumonisin (FUM), fusaric acid (FU) and apicidin F (APF) under the optimal conditions. It was noteworthy that FU, APF, and FUS were produced even under non-favorable conditions, which indicated that overexpression of *FfSGE1* could override nitrogen regulation [155]. Deletion of *sge1* in *F. verticillioides* also led to decreased production of fumonisins including FB_1_, FB_2_ and FB_3_ [156].

Transcription factor ART1, a predicted Zn(II)_2_Cys_6_ zinc finger TF, mediated starch hydrolysis and mycotoxin production in *F. graminearum* and *F. verticillioides*. ART1 played an important role in the production of both trichothecene and fumonisin by the regulation of genes involved in starch hydrolysis [157].

*FgStuA* in *F. graminearum* was a TF gene that shared homology with key developmental regulators in fungi. The deletion mutant Δ*FgStuA* significantly reduced the pathogenicity on wheat heads and the production of SMs. The production of red pigment aurofusarin was decreased in the Δ*FgStuA* mutant. The ability of the Δ*FgStuA* mutant to synthesize 15-AcDON and DON was also decreased [158]. Further investigation showed that the TF FgStuA regulated virulence and mycotoxin biosynthesis via recruiting the SAGA complex in *F. graminearum* [159].

The transcription factor FoAce2 (encoding *F. oxysporum* angiotensin converting enzyme 2) was found to regulate vegetative growth, virulence, conidiation, and cell wall homeostasis of *F. oxysporum* f. sp. *cubense.* In the Δ*FoAce2* mutant, three biosynthesis genes of BEA were down-regulated, resulting in a decrease in BEA production [160].

C_2_H_2_ was the most common TF in the zinc finger TF family, widely conserved from single-celled organisms to higher mammals [161,162]. *F. oxysporum* f. sp. *lycopersici* C_2_H_2_ TF *FolCzf1* was needed for the production of conidia and fusaric acid, and early host infection. Compared with those of WT and Δ*FolCZF1*-C strains, the Δ*FolCZF1* strain showed a significant decrease in FA production, indicating that FolCZF1 was involved in the biosynthesis of FA. In addition, under favorable conditions for FA production, the expression level of FA biosynthesis genes in *F. oxysporum* f. sp. *lycopersici* was significantly reduced, which further supported the role of FolCzf1 in regulating FA production [163].

Fp487 was a Zn_2_Cys_6_ transcription factor in *F. pseudograminearum*. Compared with the wild-type strain CF14047, the conidiation, pathogenicity, and production of 3-AcDON of the Δ*Fp487* mutant significantly decreased [164].

*ZRF1* was a zinc binuclear cluster-type gene in *F. verticillioides*. The gene *ZFR1* deletion mutant exhibited normal growth and development on maize kernels, but the production of fumonisin was decreased to less than 10% of that of the wild-type strain. Overexpression of *ZFR1* in Δ*Fvzfr1* mutant restored FB1 production to wild-type levels, which indicated that ZFR1 was a positive regulator of FB1 biosynthesis in *F. verticillioides* [165].

The HAP complex was a conserved, heterotrimeric transcriptional regulator that bound the consensus sequence CCAAT to modulate gene expression in *F. verticillioides*. The Hap3 subunit linked the HAP complex to regulate fumonisin biosynthesis. Deletion of *HAP3* suppressed fumonisin biosynthesis [166].

The gene *hxk1* was a putative hexokinase-encoding gene to modulate carbon catabolism, sporulation, FB1 production and pathogenesis in *F. verticillioides*. The Δ*hxk1* mutant produced about 50% and 80% less trehalose than the WT strain, respectively [167].

The gene *FvatfA* from the maize pathogen *F. verticillioides* putatively encoded bZIP-type transcription factor FvAtfA, which was homologous to the *Aspergillus nidulans* AtfA and *Schizosaccharomyces pombe* Atf1. Deletion of *FvatfA* led to the overproduction of bikaverin and abolishment of fumonisin production in the Δ*FvatfA* strain [168].

MADS-box TFs played a role in virulence, and vegetative and sexual development of *F. verticillioides*. Two MADS-box TFs, Mads1 and Mads2, in terms of their roles in secondary metabolism and sexual mating. The *MADS1* and *MADS2* knockout mutants exhibited decreased vegetative growth and FB1 production when compared to the wild-type strain. Mads1 was a broad regulator of secondary metabolism in *F. verticillioides*, and might target regulons upstream of Mads2 to affect FB1 production [169].

FvOshC was identified as the specific protein that bound to ergosterol in *F. verticillioides*. Gene knockout complementation techniques confirmed that FvOshC acted as a global regulatory protein to play a positive role in the pathogenicity and FB1 biosynthesis in *F. verticillioides* [170].

*Fusarium* sp. was an endophytic fungus isolated from the ambrosia beetle *Xylosandrus morigerus*. FspTF was a member of the fungal-specific family of transcription factor KilA-N/APSES. The deletion mutant Δ*Fsptf* could not synthesize the pigments javanicin and fusarubin which indicated that FspTF positively regulated pigment production [171]. Some examples of the miscellaneous TFs regulating SM production in *Fusarium* fungi are displayed in Table 5.

## 4. Epigenetic Regulation on *Fusarium* SM Production

Epigenetic regulation on SM production in fungi mainly includes modifications of histone, DNA and RNA. DNA in chromatin is organized in an array of nucleosomes. Two copies of each histone protein subunit, including H2A, H2B, H3, and H4, are assembled into an octamer surrounding by DNA of 145 to 147 base pairs, forming the core of the nucleosome [172].

Chromatin structure is the basis for regulating gene expression. The loose structure of euchromatin is associated with transcriptional activity, while the tight structure of heterochromatin is related to transcriptional repression. The epigenetic regulatory mechanisms mainly include histone acetylation, histone methylation, recognition by reader modules, sumoylation, phosphorylation, ubiquitylation, and DNA methylation, which are involved in the control of DNA expression [45].

Histone acetylation modification is controlled by two classes of enzymes known as histone acetyltransferases (HATs) and histone deacetylases (HDACs). HATs catalyze the transfer of acetyl groups from acetyl-CoA to the ε-amino group on the side chain of lysine residues of the core histones, typically forming a part of the complex [173]. On the contrary, HDACs remove acetyl moieties from lysine residues at histone tails and nuclear regulatory proteins, and thus significantly affect chromatin remodeling and transcriptional regulation in eukaryotes. Histone methylation modification is regulated by histone methyltransferases (HMTs) and histone demethylases (HDMs), which are used to add and remove methyl groups on lysine and arginine residues, respectively. So the gene expression is regulated through the synergistic effect of HATs and HDACs, or HMTs and HDMs [22].

Chromatin modifications and heterochromatic labeling are associated with the regulation of fungal secondary metabolism BGCs [174]. In *Fusarium* fungi, some epigenetic regulation strategies have been reported to regulate secondary metabolism including the regulations of HATs, HDACs, HMTs, HDMs, chromatin readers, DNA methylation, and RNA modifications. Epigenetic regulation on *Fusarium* SM production should be an efficient strategy by activating useful metabolite production or inhibiting toxic metabolite production [21,22,175,176].

### 4.1. Regulation of HATs

Histone acetylation by HATs leads to activation of euchromatin to positively regulate gene expression of fungal secondary metabolism [22].

In the Δ*elp3* mutant of *F. graminearum*, the amount of perithecia formed was reduced and maturation of perithecia was delayed.

The main trichothecenes such as DON and 15-AcDON were not detected in the Δ*Fgelp3* mutant, while both DON and 15-AcDON were produced at detectable levels in the WT strain. The RT-qPCR analysis showed that transcription of the trichothecene biosynthesis genes *Fgtri5* and *Fgtri6* was significantly reduced in the Δ*Fgelp3* mutant compared with the WT strain. In a virulence test, 21 days after wheat head inoculation, the disease symptoms caused by the Δ*elp3* mutant were significantly reduced, while the wild-type and complementary strains caused typical wheat blight symptoms. This indicated that the HAT gene *Fgelp3* was involved in various biological processes, including sexual and asexual reproduction, SM production, and virulence in *F. graminearum* [177].

DON was a mycotoxin produced by *Fusarium* species. This mycotoxin was a virulence factor that assisted fungi in colonizing and spreading within spikes. DON, originally known as vomitoxin, has severe vomiting effects on some animals such as humans, pigs, dogs and minks [178]. In *F. fujikuroi*, the Δ*FfGcnE* mutant showed an obvious deficiency of H3K9/K14/K27ac and the reduced production of 18 metabolites [179]. The deletion of *FgGcnE* in *F. graminearum* led to decreased production of DON [180,181,182]. In addition, the deletion of the bromodomain of *FgGcn5* led to a significant reduction in DON production and virulence of *F. graminearum* [183].

The deletion of *FgSas3* reduced production of DON, and reduced sporulation and perithecium formation, which demonstrated that Sas3 positively regulated DON biosynthesis and sporulation in *F. graminearum* [180].

Deletion of *hat1* resulted in downregulation of GA gene expression and decreased GA production in *F. fujikuroi*. Instead, overexpression of *hat1* resulted in an upregulation of GA gene expression and an increase in the production of GAs [104].

Although the *Fghat1* deletion mutant (Δ*Fghat1*) of *F. graminearum* was normal in fungal growth, asexual and sexual development, and pathogenicity, it had severe defects in DON production. Exogenous cAMP treatment rescued the defects of the Δ*Fghat1* mutant in DON production, indicating a relationship between FgHat1 and cAMP signaling in *F. graminearum* [184].

The Δ*Fghat2* mutant of *F. graminearum* produced significantly decreased levels of DON compared to the wild-type strain. Compared with the wild-type strain, the expression levels of Tri6 and Tri12 in the Δ*Fghat2* mutant were significantly reduced, indicating that *FgHAT2* was essential for DON biosynthesis in *F. graminearum* [185]. Some examples of HATs regulating SM production in *Fusarium* fungi are displayed in Table 6.

### 4.2. Regulation of HDACs

Histone deacetylation by HDACs leads to the formation of heterochromatin and suppresses gene expression. So HDACs negatively regulate gene expression of fungal secondary metabolism [22,45]. HDACs regulating SM production in *Fusarium* fungi included class I HDACs (i.e., Hda2, Hos2, and Rpd3), class II HDACs (i.e., HdaA, Hdf1, and Hdf2) and class III HDACs (i.e., Hst2, Sir2, and SirD).

#### 4.2.1. Regulation of Class I HDACs

If the gene *Ffhda2* in *F. fujikuroi* was deleted, the production of bikaverin, fusarubin, fusaric acid, and gibberellins (GAs) including GA3, GA4 and GA7 was decreased. It was estimated that FfHda2 was necessary for the virulence of *F. fujikuroi* by regulating the production of SMs in rice seedlings [186].

An increase in H4K16ac levels was observed in the *Fvhos2* deletion mutant of *F. verticillioides*. The fumonisin B1 production was decreased in the ∆*Fvhos2* strain, which meant FB1 biosynthesis was positively regulated by FvHos2. Accordingly, in the ∆*Fvhos2* strain, the expression of *FUM* genes such as *FUM1*, *FUM8*, *FUM19*, and *FUM21*, was significantly reduced [35].

Overexpression of *FvRpd3* in *F. verticillioides* increased FB1 production. Therefore, *FUM* genes such as *FUM1*, *FUM8*, *FUM19*, and *FUM21*, showed significantly high expression in the *FvRpd3*-OE strain [35].

#### 4.2.2. Regulation of Class II HDACs

If the gene *Hda1* was deleted in *F. fujikuroi*, the production of BEA would increase by 1000 times. However, the deletion of the gene *Ffhda1* in *F. fujikuroi* led to a decrease in the production of plant hormones GAs including GA3, GA4 and GA7, as well as a decrease in the production of bikaverin, fusarubin and fusaric acid. Only the production of fusarin A was increased. It was estimated that FfHda1 was required for the virulence of rice seedlings [187].

Deletion of *Fvhda1* in *F. verticillioides* increased in the production of FB1, indicating that FvHda1 negatively regulated the biosynthesis of fumonisins. In addition, the RT-qPCR revealed an increase in *FUM1* expression in the Δ*Fvhda1* mutant [35].

The absence of *Fghdf1* significantly reduced the virulence and DON production of *F. graminearum*. Furthermore, the ∆*Fghdf1* mutant had stronger tolerance to H_2_O_2_ than the WT strain [188].

If the gene *Fahdf2* was deleted in *F. asiaticum*, the production of 4-AcNIV and 4,15-diAcNIV would increase [189].

#### 4.2.3. Regulation of Class III HDACs

Class III HDACs belong to sirtuin type NAD^+^-dependent deacetylases whose activities are sensitive to intracellular NAD^+^ availability [190]. Class III HDACs including Hst2 (HstB, SirT2), Hst4 (SirT4), SirT5, Hst4, SirA, Sir2, SirD (Sir4), and SirE have been reported to regulate fungal secondary metabolism [22].

Hst2 was also named HstB or SirT2. If the gene *Fvhst2* was deleted in *F. verticillioides*, the causal agent of destructive diseases of maize, the level of H4K16ac was increased. Correspondingly, the production of FB1 was increased, which indicated that the FB1 biosynthesis was negatively regulated by *Fvhst2*. In addition, when sugarcane and maize were infected with the ∆*Fvhst2* mutant, the vegetative growth, conidiation, and virulence of the mutant were increased [35].

When the gene *Fvsirt4* was overexpressed in *F. verticillioides*, FB1 production was decreased. Accordingly, the key *FUM* genes (i.e., *FUM1*, *FUM8*, and *FUM19*) involved in FB1 toxin synthesis were significantly decreased in the *FvSirt4*-OE strain [35].

SirT5 and Sir2 were involved in histone acetylation at H3K9, H3K14, H3K27, and H4K16 residues in fungi. Fumonisin B production was significantly reduced in the Δ*Fvsirt5* mutant of *F. verticillioides*, and the expression of genes (*FUMs* and *PKSs*) involved in secondary metabolism was also significantly down-regulated [191].

Sir2 was known as SirB. The absence of *Fvsir2* in *F. verticillioides* led to an increase in FB1 production, which indicated that FvSirB negatively regulated fumonisin biosynthesis [35]. However, fumonisin B production was significantly reduced in the Δ*sir2* mutant of *F. verticillioides*, the expression of genes related to the biosynthesis of fumonisin was also significantly downregulated [191]. Some examples of HDACs regulating SM production in *Fusarium* fungi are shown in Table 7.

### 4.3. Regulation of HMTs

Histone methylation is the process of primarily adding methyl groups from *S*-adenosyl-1-methionine (SAM) to lysine or arginine residues through histone methyltransferases (HMTs). Histone methylation leads to chromatin tightening, making it difficult for related gene regions to be bound by TFs, RNA polymerases and other proteins, thereby inhibiting gene transcriptional activity. Therefore, HMTs negatively regulate gene expression of fungal secondary metabolism [192].

H3K27me3 was a major histone post-translational modification (PTM) in *F. fujikuroi*. Deletion of the methyltransferase Kmt6 reduced the burden of H3K27me3 and resulted in the induction of cryptic and silent SM BGCs in *F. fujikuroi*. One of the four putative SM BGCs, named STC5, was analyzed in more detail thereby revealing a new sesquiterpene (1*R*,4*R*,5*S*)-guaia-6,10(14)-diene [193]

The deletion of H3K27me3 had a more significant impact on the expression of SM BGCs than the intensely studied regulation by nitrogen. Deletion of *kmt6* led to production of mycotoxins, pigments and other SMs in *F. graminearum* [194].

Lack of COMPASS component Ccl1 reduced H3K4 trimethylation levels and affected biosynthetic gene transcription and production of gibberellic acid in *F. fujikuroi*, and biosynthetic gene transcription and production of DON in *F. graminearum* [195].

Set2 and Ash1 were two HMTs targeting H3K36 in different chromatin regions in *F. fujikuroi*. In addition to HMT activity, Set2 also interacted directly with the elongation form of RNAPII through its SRI domain to activate gene transcription. H3K36me mediated by Ash1 in the sub-telomeric regions could inhibit the HMT activity of PRC2 on H3K27 and prevent the formation of heterochromatin. In addition, Set2 and Ash1 controlled the expression of multiple TFs and histone modifier-encoding genes, indirectly regulating the production of metabolites [196].

The methylation of lysine 20 of histone 4 (H4K20me) in *F. fujikuroi* and *F. graminearum* was functionally characterized. *FfKMT5* in *F. fujikuroi* and *FgKMT5* in *F. graminearum* were identified as solely responsible for H4K20 mono-, di- and trimethylation. The deficiency of Kmt5 had a significant impact on the secondary metabolism in two plant pathogens *F. fujikuroi* and *F. graminearum* with the most positive regulation on the biosynthesis of fusarin C in *F. fujikuroi* and zearalenone biosynthesis in *F. graminearum* [197].

FgSet1 was mainly responsible for monomethylation, dimethylation and trimethylation of H3K4 in *F. graminearum* [198]. The *FgSet1* deletion mutant (Δ*FgSet1*) was impaired in hyphal growth and virulence. H3K4me was required for the active transcription of genes involved in DON and aurofusarin biosynthesis. Deletion of *FgSet1* decreased production of DON and aurofusarin, which indicated that *FgSet1* positively regulated biosynthesis of DON and aurofusarin [199].

Disruption of H3K27 methylation via Δ*kmt6* mutants of *F. graminearum* led to the production of a cyclic lipopeptide fusaristatin A. Overexpression of the gene *kmt6* led to the production of three pyrone derivatives gibepyrone A, and fusapyrones A and B, which highlighted the role of chromatin remodeling in metabolic diversity [200].

The post-translational trimethylation of histone 3 lysine 9 (H3K9me3) was considered a marker of heterochromatin, and was established by the SET-domain protein Kmt1. FmKmt1 participated in H3K9me3 in *F. mangiferae*. The absence of *FmKmt1* significantly affected fungal growth and stress response, which was essential for wild-type-like conidiation. Although *FmKmt1* was essentially unnecessary for the biosynthesis of most known SMs, the deletion of *FmKmt1* resulted in an almost complete loss of fusapyrone and deoxyfusapyrone [201].

*F. proliferatum* was a member of the *F. fujikuroi* species complex (FFSC). In the wild-type strain, the deletion of *Fpkmt6* encoding the H3K27-specific histone methyltransferase resulted in the elevated expression of 49% of genes. However, the genes involved in the biosynthesis of the gibberellins (GAs) were among the most upregulated genes in the Δ*Fpkmt6* mutant. This indicated that H3K27me3 was involved in GA gene expression in *F. proliferatum*. The H3K27me3-specific methyltransferase FpKmt6 was the key regulator that inhibited the expression of secondary metabolism genes in *F. proliferatum* [32].

The deletion of *FvSet1* in *F. verticillioides* led to various defects in fungal growth and pathogenicity. Furthermore, the Δ*FvSet1* mutant exhibited a significant defect in FB1 biosynthesis with lower expression levels of FUM genes. FvSet1 played an important role in *F. verticillioides* in the responses to various environmental stresses by regulating the phosphorylation of FvMgv1 and FvHog1 [202].

FvSet2 in *F. verticillioides* was an ortholog of *S. cerevisiae* Set2. FvSet2 was responsible for the trimethylation of histone 3 lysine 36 (H3K36me3). The Δ*FvSet2* mutant exhibited significant defects in vegetative growth, FB1 biosynthesis, pigmentation, and fungal virulence. In addition, trimethylation of H3K36 was important for the active transcription of genes related to the biosynthesis of FB1 and bikaverin [203]. Some examples of HMTs regulating SM production in *Fusarium* fungi are shown in Table 8.

### 4.4. Regulation of HDMs

Histone demethylation by HDMs leads to methyl groups being removed from lysine and arginine residues of histone. The chromatin is activated. So HDMs positively regulate gene expression of fungal secondary metabolism.

There are a few reports about the regulation of HDMs on fungal secondary metabolism. FgKdm5 in *F. graminearum* was a homolog of KDM5 proteins belonging to the diverse JmjC domain-containing superfamily. It had the function of the histone demethylase. Lack of *FgKdm5* resulted in a significant decrease in the production of five SMs including DON, fusarin C, zearalenone, fusarielin H and chrysogine, which indicated that *Fgkdm5* positively regulated SM production in *F. graminearum* [204].

### 4.5. Regulation of Chromatin Readers

The bromo-adjacent homology (BAH)-plant homeodomain (PHD) containing protein BAH–PHD protein 1 (BP1) was a reader for H3K27 methylation in *F. graminearum*, which was the cereal fungal pathogen. BP1 interacted with the core polycomb repressive complex 2 (PRC2) component Suz12 and directly bound methylated H3K27. BP1 was distributed in a subset of genomic regions marked by H3K27me3 and co-repressed gene transcription. The BP1 deletion mutant showed the same phenotypes in fungal growth and virulence. The expression profile of secondary metabolism genes was similar to that of strains lacking H3K27 methyltransferase Kmt6. Furthermore, BP1 could directly bind to DNA through its PHD finger, which might increase the residence of nucleosomes and enhance transcriptional inhibition of H3K27me3-labeled target regions. So BP1 negatively regulated secondary metabolism. BP1 was considered a novel methylated H3K27 reader that played important roles in fungal development and pathogenicity, as well as the production of SMs. Its orthologs were widely distributed in ascomycetes, indicating that the compounds which actively targeted BP1 could be used to manage plant fungal diseases, especially those caused by *Fusarium* species [27].

### 4.6. Regulation of DNA Methylation

DNA methylation plays important roles in eukaryotic gene expression and silencing, cell differentiation, and phylogeny. It may lead to changes in chromatin structures, DNA stability, and DNA-protein interactions, thereby affecting gene expression [205].

FgDIM-2 and FgRID were two DNA methyltransferases (DNMTs) in *F. graminearum*. FgDIM-2 was a homologue to DIM-2 (deficient in methylation) from *N. crassa*, and FgRID was a homologue to RID (repeat-induced point (RIP) deficient) from *N. crassa*. The production of 15-AcDON was increased by the dual-deletion strain Δ*FgDim-2*Δ*FgRid*, which indicated that the DNMTs negatively regulated 15-AcDON production [206].

### 4.7. Regulation of RNA Modifications

The regulation of RNA modifications mainly includes RNA methylation, RNA interference, and non-coding RNA. Two non-coding RNAs (ncRNAs) including long non-coding RNA (lncRNA) and small interfering RNA (siRNA), were found to regulate secondary metabolism in *Fusarium* fungi.

Both *FgTri5* expression and DON biosynthesis were regulated by a long non-coding RNA (lncRNA), namely RNASP in *F. graminearum*. By replacing the promoter region of *Tri5* with the promoter region of *Tri12* to delete *RNA5P*, the expression of *Tri5* and the biosynthesis of DON were increased [207].

Small interfering RNA (siRNA) mediated gene silencing led to the overproduction of bikaverin in *Fusarium* sp. HKF15 [208].

### 4.8. Miscellaneous Epigenetic Regulation

Miscellaneous epigenetic regulations include chromatin remodeling, histone phosphorylation, ubiquitylation, and sumoylation. In *F. graminearum*, if the genes *Msg5* and *Yvh1* of phosphatases were deleted, the production of DON was decreased, indicating the complex regulatory networks were involved in the production of *Fusarium* SMs [209].

Transcription activator FgDDT interacted with the chromatin remodeling factor FgISW1 to regulate the development and pathogenicity of *F. graminearum* [210].

FvHP1 was a speculated member of the heterochromatin protein 1 (HP1) family in *F. verticillioides*. FvHP1 retained the essential residues required for H3K9me2/3 recognition. Phenotypic analysis of the ∆*FvHP1* mutant showed impaired vegetative growth, reduced conidiation and virulence, and altered FB1 production. In addition, the accumulation of red pigments (i.e., aurofusarin) in the mutant was linked to the deregulation of secondary metabolism, specifically the overproduction of fusarubin-type naphthoquinones, such as 8-*O*-methyl nectriafurone in *F. verticillioides* [211].

## 5. Regulation of Signal Transduction on *Fusarium* SM Production

Some signal transduction pathways have been revealed to regulate *Fusarium* SM production including the cAMP signaling pathway, TOR signaling pathway, MAPK signaling pathway, and G protein signaling pathway [23].

### 5.1. Regulation of cAMP Signaling Pathway

The cAMP signaling pathway (or called the cAMP pathway) includes regulatory subunits of cAMP-dependent protein kinase (PKA) to investigate their roles in fungal sexual development, colony structure, and the regulation of secondary metabolism.

The cAMP pathway had an impact on GA and bikaverin biosynthesis. cAMP inhibited fusarubin biosynthesis in *F. fujikuroi* [212].

The absence of the catalytic domain of the AcyA protein in *F. fujikuroi* resulted in various phenotypic changes, such as slower mycelial growth, increased synthesis of red pigments (i.e., aurofusarin), decreased synthesis of gibberellins, and partial activation of carotenoid biosynthesis even under dark conditions [213].

The biosynthesis of fusarin was subject to a complex control including regulators from diverse signaling pathways [214]. The gene *FfacyA* encoded an adenylate cyclase. It linked light regulation to cAMP signaling. AcyA was a positive regulator of fusarin biosynthesis in *F. fujikuroi* [214]. The gene *carS* encoded a RING finger protein repressor in *F. fujikuroi* [215]. It is involved in carotenoid regulation. CarS was a positive regulator of fusarin biosynthesis in *F. fujikuroi* [214]. The gene *FfwcoA* encoded a white collar photoreceptor in *F. fujikuroi*. It linked light regulation to cAMP signaling. WcoA was a positive regulator of fusarin biosynthesis [214].

*F. graminearum* contained two genes, *FgCPK1* and *FgCPK2*, which encoded the catalytic subunits of cAMP-dependent protein kinase A (PKA). The deletion of *cpk1* led to a marked reduction in conidiation, vegetative growth and DON biosynthesis, while simultaneously enhancing the fungal tolerance to elevated temperatures [216].

### 5.2. Regulation of TOR Signaling Pathway

The target of rapamycin (TOR) protein is a key signal-transducing component that regulates cell growth and metabolism. In the *F. fujikuroi* genome, a single TOR-encoding gene has been identified, and it was essential for viability. Through pharmacological inhibition, TOR disruption led to widespread disturbances in cellular function. This included the dysregulation of AreA-mediated secondary metabolism and a strong downregulation of genes associated with signal transduction, ribosome formation, protein translation and autophagy. Interestingly, when TOR was inhibited by rapamycin under nitrogen-limited conditions, the nitrogen catabolite repression was partially lifted, suggesting a modulatory role of TOR in nitrogen sensing. Despite this, the exact involvement of TOR in nitrogen regulation remained poorly understood [217]. Genome-wide analyses revealed that not all TOR-responsive genes were controlled by the transcription factor AreA, indicating a sophisticated regulatory network where both the glutamine synthetase (GS) and TOR pathways competed and collaborated [124,218]. While rapamycin-induced activation of GS occurred independently of AreA, the downstream targets of GS were indirectly and inversely affected by TOR inhibition. Nevertheless, both signaling routes converged to support GA_3_ production [47]. The precise mechanism in *F. fujikuroi* needs further investigation [15].

### 5.3. Regulation of the MAPK Signaling Pathway

Mitogen-activated protein kinase (MAPK) pathways are crucial regulators of secondary metabolism in fungi. In *F. graminearum*, the involvement of MAPK cascades in the biosynthesis of trichothecenes, a class of toxic SMs that have been widely investigated, highlighting their significant role in modulating the expression and activity of genes associated with toxin production [100].

In standard laboratory culture conditions, the absence of *MK1* (*MAP kinase 1*) in *F. verticillioides* resulted in lower expression levels of key biosynthetic genes *FUM1* and *FUM8*, along with a significant reduction in fumonisin accumulation. Restoration of the *Fvmk1* mutant by reintroducing the wild-type *FvMK1* gene reversed these effects, demonstrating that *FvMK1* played a positive regulatory role in fumonisin biosynthesis [219].

The deletion of *Fphog1*, a HOG-type MAP kinase encoding gene in *F. proliferatum*, was found to enhance both FB1 production and the expression of *FUM* genes when grown under nitrogen-limited conditions [220]. This suggested that MAPK signaling pathways were involved in modulating FB1 biosynthesis in response to environmental factors, particularly nutrient availability.

The elevated pH levels have been shown to enhance FB production in *F. proliferatum* through activation of the MAPK pathway [138]. Furthermore, the disruption of *FvBCK1,* a gene encoding a MAP kinase that acted as the primary upstream element of the cell wall integrity (CWI), resulted in reduced FB1 biosynthesis compared to the WT strain, indicating that the CWI MAPK pathway played a critical role in regulating FB1 production in *F. proliferatum* [221].

### 5.4. Regulation of Other Signaling Pathways

GAC1, a GTPase-activating protein, enhanced bikaverin production in *F. verticillioides*. It demonstrated a critical role of the signal transduction pathway in regulating bikaverin biosynthesis [222].

In *F. verticillioides*, *FvMK1* was a mitogen-activated protein kinase gene that played a pivotal role in fungal development and virulence. Deletion of *FvMK1* resulted in a complete loss of pathogenicity, with the mutant unable to colonize host tissues through wound sites. Moreover, it failed to induce stalk rot symptoms beyond the point of inoculation on corn stalks, highlighting the essential role of genes during the process of infecting plants. The Δ*Fvmk1* mutant also exhibited a marked reduction in fumonisin production along with significantly lower expression of *FUM1* and *FUM8,* two key genes in the fumonisin biosynthetic pathway. These findings underscored the importance of *FvMK1* in regulating multiple biological processes, including vegetative growth, asexual sporulation, mycotoxin production, and pathogenicity of *F. verticillioides* [219].

G protein signaling in *F. fujikuroi* played a critical role in fungal growth, secondary metabolism and sexual development. Specifically, Gα subunits FfG1 and FfG3 acted as the negative regulators of fusarubin biosynthesis [212].

## 6. Regulation of Organic Chemicals and Plant/Microorganism-Derived Extracts on *Fusarium* SM Production

Organic chemicals, which are known as low molecular weight organic compounds, can regulate the secondary metabolism of fungi [21]. Some organic chemicals are synthesized, while others are natural and derived from plants and microorganisms. Most chemicals are considered epigenetic modifiers, and other chemicals function as signaling compounds, precursors, inhibitors of SM biosynthesis. Furthermore, the regulatory functions of some chemicals remained unclear [21,223]. Currently, these studies were only conducted in vitro. The regulations of the chemicals with known structures, along with the plant/microorganism-derived extracts with the compound structures unclear on *Fusarium* SM production, are introduced as follows.

### 6.1. Regulation of Organic Chemicals

The production of both 4-aminobutyrate (GABA) and DON was increased in *F. asiaticum* by adding 2 mM agmatine. GABA might be biosynthesized from agmatine through putrescine as the intermediate, and DON biosynthesis was also influenced [224].

Compactin (known as 6-demethylmevinolin or 6-DMM), an HMG-CoA reductase inhibitor produced by *Penicillium citrinum*, was involved in the regulation of cholesterol biosynthesis. This compound has been shown to effectively suppress the biosynthesis of the polyketide mycotoxin aflatoxin B1 (AFB1) in *Aspergillus flavus*. Compactin also inhibited melanin synthesis and blocked spore development [225]. Furthermore, compactin suppressed the production of DON and ZEN in *F. culmorum* at 25 μg/mL in medium [226].

6-Pentyl-α-pyrone (6PAP) was an antifungal compound produced by the biological control fungus *Trichoderma* sp. The presence of 6PAP in the culture medium led to a significant reduction in DON production by *F. graminearum* [227].

Two plant-derived lignans, pinoresinol and secoisolariciresinol, showed inhibitory activity on mycelia growth and trichothecene biosynthesis in *F. graminearum*. Both pinoresinol and secoisolariciresinol at concentrations of 1.25 mg/L and 5.0 mg/L in the medium all inhibited radial growth and decreased production of DON and nivalenol (NIV) in *F. graminearum*. RT-qPCR analysis revealed that ligan treatment reduced trichothecene production in *F. graminearum* linked with downregulation of mRNA expression of the genes *tri4*, *tri5* and *tri11* [228].

The production of DON in *F. graminearum* was inhibited with 0.5 mM of caffeic acid supplementation, although the fungal growth was not affected at this dosage (0.5 mM) applied to the fungus [229]. NPD12671 was a synthetic furanocoumarin derivative. It stimulated production of 15-AcDON at 2 μM in *F. graminearum*. However, dihydroartemisinin (DHA) was screened to inhibit the production of 15-AcDON at 5 μM in *F. graminearum*. It was found that NPD12671 stimulated trichothecene production through activation of *Tri6* expression, and DHA inhibited trichothecene production through repression of *Tri6* transcription [230]. Nicotinamide at 500 µg/mL markedly inhibited the synthesis of DON and ergosterol peroxide in *F. graminearum*, the fungal pathogen responsible for wheat head blight [231].

The mycelia of *F. graminearum* were grown on PDB supplemented with two concentrations (3 µg/mL and 10 µg/mL) of trichostatin A (TSA) for 48 h, 72 h, and 96 h, respectively. After which, the mRNA levels were approximated via RT-qPCR analysis. It was then found that the HADC levels and trichodiene synthase gene *Tri5* were different over time and the amount in response to the use of TSA. Treatment with TSA induced upregulation of *Tri5* gene expression in the toxigenic isolate, with the obvious expression observed after 48 h at 3 µg/mL [232].

The histone acetyltransferase Gcn5 played a crucial role in epigenetic regulation. Phenazine-1-carboxamide (PCN) decreased DON production by inhibiting FgGcn5 in *F. graminearum*. The molecular mechanism of FgGcn5 inhibition by PCN was also unraveled by combined in silico and in vitro investigations [233]. 2-Hydroxy-4-methoxybenzaldehyde (HMB) was a SM with antimicrobial activity in many plant species [234]. The treatment of HMB at the minimum inhibitory concentration (MIC, 100 μg/mL) notably decreased the production of ergosterol and DON biosynthesis in *F. graminearum*. RT-qPCR investigation showed that the HMB treatment importantly modulated the expression of the key genes, including those of ergosterol biosynthesis (*Erg2*, *Erg5*, *Erg6*, etc.), DON biosynthesis (up to 16 genes), global regulators (*LaeA*, *VeA*, and *VelB*), the redox system (i.e., *MnSOD*, *Cu*/*ZnSOD*, *GSS*, and *CAT*), and stress signaling pathways (i.e., *Hog1*, *Ssk1*, *Ssk2*, and *Pbs2*) [234]. Exposure of *F. graminearum* to citric acid at 5 or 10 mM led to a marked decrease in biosynthesis of type B trichothecene mycotoxins, including DON, 3-AcDON, 15-AcDON, and NIV. However, the mycelial growth and pigment biosynthesis were enhanced [235].

The endophytic fungus *F. oxysporum* was treated with epigenetic modifier prednisone (300 μM), and the production of active compound umbelliferone was increased [236].

Treatment of *F. oxysporum* f. sp. *conglutinans* with 500 µM suberoyl bis-hydroxamic acid (SBHA) led to the induction of secondary metabolic pathways, resulting in the production of two novel fusaric acid derivatives, 5-butyl-6-oxo-1,6-dihydropyridine-2-carboxylic acid and 5-(but-9-enyl)-6-oxo-1,6-dihydropyridine-2-carboxylic acid [237].

The effects of different chemical modifiers, including 5-azacytidine (5-Aza), nicotinamide (NIC), sodium butyrate (SB), and sodium valproate (SV) on the metabolic profiles of *F. verticillioides* were assessed. After treatment, the fungal metabolome was analyzed using UHPLC–HRMS/MS in both untargeted and targeted metabolomics modes. The most pronounced changes in secondary metabolism were observed with SV, which significantly altered the metabolic fingerprint of *F. verticillioides*, likely through the activation of silent or cryptic biosynthetic gene clusters. Multivariate analysis highlighted 50 metabolites that most distinctly separated the five treatment conditions. Of these, twelve were annotated as fusarins or structural analogs. In comparison, NIC and SB induced only minor changes in the production of these compounds, indicating a weaker modulatory effect on the fungal secondary metabolism under the tested conditions [238].

Linoleate diol synthase 1 (LDS1) primarily generated 8-hydroperoxyoctadecenoic acids, which were further converted into various di-hydroxyoctadecenoic acids. The *Fvlds1*-deleted mutant of *F. verticillioides* exhibited enhanced growth, increased conidiation, higher fumonisins production, and faster maize cobs infection compared to the WT strain, which indicated that oxylipins produced by LDS1 acted as negative promoters of growth, conidiation, and fumonisin biosynthesis in *F. verticillioides*, a maize fungal pathogen [239].

The exposure of *Fusarium* sp. RK97-94 to the protein synthesis inhibitor hygromycin B, was found to induce the biosynthesis of several SMs, including lucilactaene, NG-391, fusarubin, 1233A, and 1233B. 1233A was identified as an inhibitor of HMG-CoA synthase. Genomic analysis led to the identification of the biosynthetic gene cluster responsible for 1233A production, which comprised four key genes. Notably, one of these genes played a role in providing self-resistance to the producing organism, protecting it from the inhibitory effects of 1233A [240].

The treatment of *Fusarium* sp. RK97-94 cultures with 30 µM NPD938 triggered the biosynthesis of three derivatives of lucilactaene including dihydroNG391, dihydrolucilactaene, and 13α-hydroxylucilactaene. Evaluation of their antimalarial potential against *Plasmodium falciparum* revealed significant differences in activity. DihydroNG391 showed only modest efficacy with an IC_50_ of 62 µM, whereas dihydrolucilactaene demonstrated strong potency with an IC_50_ of 0.0015 µM, and 13α-hydroxylucilactaene exhibited intermediate activity with an IC_50_ of 0.68 µM. The structure–activity relationship studies indicated that the absence of the epoxide moiety, specifically, its reduction in NG391 to yield dihydrolucilactaene which led to a dramatic 1200-fold enhancement in antimalarial activity, highlighting the negative impact of the epoxide group on potency. Furthermore, opening the tetrahydrofuran ring in 13α-hydroxylucilactaene to generate dihydrolucilactaene resulted in a 100-fold increase in activity, suggesting that the integrity of the pyrrolidone ring and the lack of an epoxide are more crucial for antimalarial efficacy than the presence of the tetrahydrofuran ring. In cytotoxicity assays, dihydrolucilactaene showed low toxicity toward human cancer cell lines, with IC_50_ values of 21 µM against HeLa cells and 37 µM against HL-60 cells [241]. Some examples of organic chemicals to regulate SM production in *Fusarium* fungi are displayed in Table 9.

### 6.2. Regulation of the Extracts from Plants and Microorganisms

The crude extracts were prepared from plants, bacteria and fungi. There were multiple chemicals (components) with their structures unknown in each crude extract. The regulatory mechanisms of the extracts from plants and microorganisms should be complex.

#### 6.2.1. Regulation of Plant Extracts

Putrescine was a defense compound produced by wheat could induce hypertranscription of *F. graminearum* trichothecene biosynthetic genes (*FgTRIs*), leading to increased DON accumulation during fungal infection. Further investigation into the regulatory mechanisms revealed that the transcription factor FgAreA mediated putrescine-induced *FgTRIs* expression by promoting histone modifications specifically, histone H2B monoubiquitination (H2Bub1) and histone 3 lysine 4 di-/trimethylation (H3K4me2/me3) at the FgTRIs loci. This indicated that wheat defense compound putrescine triggered mycotoxin DON synthesis by regulating H2B ub1 and H3K4me2/3 deposition in *F. graminearum* [242].

The essential oils from *Eucalyptus camaldulensis* (Myrtaceae) green branches and *Origanum majorana* (Labiatae) whole plants were screened to reduce *Tri4* gene expression and mycelia growth of *F. oxysporum* strains in vitro. The production of trichothecenes in *F. oxysporum* was also decreased. GC-MS analysis showed that 1,8-cineole and spathulenol were the main compounds in the essential oil of *E. camaldulensis* green branches, and tricyclene, and *p*-cymene were the main compounds in the essential oil of *O. majorana* whole plants. Further investigation was needed to determine whether these compounds were active in affecting mycelia growth and mycotoxin production in *F. oxysporum* [243].

The regulatory mechanisms of host plant metabolites in the plant–*Fusarium* interaction have been investigated [244]. The effects of selected plant metabolites on *F. proliferatum* metabolism were analyzed. Quercetin-3-glucoside (Q-3-Glc) and kaempferol-3-rutinoside (K-3-Rut) were found to enhance fungal growth, whereas compounds such as DIMBOA (2,4-dihydroxy-7-methoxy-2H-1,4-benzoxazin-3-one), isorhamnetin-3-*O*-rutinoside (Iso-3-Rut), ferulic acid (FA), protodioscin, and neochlorogenic acid (NClA) suppressed *F. proliferatum*. The influence of these metabolites on the expression of key *FUM* genes was evaluated using RT-qPCR in liquid cultures supplemented with the compounds. Twenty-four hours after treatment, chlorogenic acid (ClA) upregulated the expression of CPR6 and SSC1, while DIMBOA and protodioscin downregulated these genes. By the third day of exposure, FUM1 transcription was increased by all metabolites except Q-3-Glc, relative to the control. FUM6 expression was induced by protodioscin, K-3-Rut, and ClA, but repressed by FA and DIMBOA. In contrast, FUM19 was upregulated by all tested metabolites except ferulic acid, highlighting the compound-specific modulation of fumonisin biosynthesis pathways in response to plant-derived chemicals [245].

Fumonisin production by *F. proliferatum* was evaluated in liquid cultures supplemented with extracts from various host plants, including pineapple fruit, white asparagus spears, garlic bulbs, six-week-old pea plants, and young maize cobs. Analysis of both mycelia and culture media revealed that asparagus extract triggered the highest increase in fumonisin (FB) levels, with garlic extract showing the second strongest stimulatory effect. Pineapple extract was particularly effective in upregulating the gene *fum1* as a key gene in the fumonisin biosynthetic pathway and significantly enhanced fumonisin synthesis across multiple fungal strains. In contrast, pea plant extract suppressed both fungal growth and mycotoxin production, indicating an inhibitory influence on *F. proliferatum* metabolism [246].

*F. proliferatum* strains PEA1 and PEA2, and *F. oxysporum* strains 34 OX and 1757 OX were two plant fungal pathogens originally identified from infected pea (*Pisum sativum*). The main metabolites were isolated from pea. It was found that coumarin, spermidine, *p*-coumaric acid, isoorientin, and quercetin (each at 100 ng/mL) reduced the growth of the fungal pathogens. All the metabolites highly inhibited the biosynthesis of FB1 and BEA [247].

#### 6.2.2. Regulation of Fungal Extracts

The production of *Fusarium* SMs was significantly influenced by fungal extracts [248]. Yeast extracts, which were rich in nitrogen-containing compounds, played a significant role in modulating the secondary metabolism of *Fusarium* species. Selected yeast strains isolated from wheat grains and bread have demonstrated the ability to suppress the production of key mycotoxins, including DON, nivalenol (NIV), and zearalenone (ZEA or ZEN), isolated from *F. culmorum*, *F. graminearum*, and *F. poae* [248]. Fusaristatin A production in *F. graminearum* was found to be highest when the fungus was cultured in yeast extract-sucrose (YES) medium. Subsequent analysis revealed that cultivation conditions significantly influenced yield, with fusaristatin A levels in stationary liquid cultures exceeding those in agitated cultures by more than fourfold, highlighting the importance of growth conditions on metabolite [249].

The influence of yeast extract on SM production was investigated in four *Fusarium* species including *F. avenaceum*, *F. fujikuroi*, *F. graminearum*, and *F. pseudograminearum*. Yeast extract significantly enhanced the synthesis of DON and zearalenone in *F. graminearum* and *F. pseudograminearum*, with certain extracts leading to high toxin levels. In *F. avenaceum*, the production of chlamydosporol, 2-AOD-3-ol, and enniatins was altered by yeast supplementation, while in *F. fujikuroi*, yeast extract influenced the biosynthesis of bikaverin, gibberellic acid, fumonisin, and fusaric acid. In contrast, the production of fusarin C and aurofusarin remained unaffected by yeast extract across all *Fusarium* strains capable of producing these metabolites, indicating a selective regulatory effect of yeast-derived components on fungal secondary metabolism [250].

#### 6.2.3. Regulation of Bacterial Extracts

The SMs (i.e., iturin A, fengycin, surfactin and bacitracin) from *Bacillus velezensis* WB induced oxidative equilibrium damage and reduced fusaric acid synthesis in *F. oxysporum* f. sp. *niveum*. The expression of fusaric acid biosynthesis-related genes was also down-regulated [251].

## 7. Other Regulations of *Fusarium* SM Production

Other regulations of *Fusarium* SM production mainly include metabolic shunting [252,253], transporters [254], and development-related proteins [255].

### 7.1. Regulation of Metabolic Shunting

Metabolic shunting was also called genetic dereplication, is a strategy that has been previously utilized to uncover novel SMs, particularly low-abundance compounds from fungi species [256,257]. The overexpression of two GA3 biosynthetic pathway genes *ggs2* and *cps/ks* enhanced 150% of GA3 production in *F. fujikuroi*. However, the overexpression of *hmgR* and *fppS* resulted in lower metabolite production, likely due to negative feedback regulation of HmgR. To overcome this, the transmembrane domains of HmgR were deleted, and the catalytic domain was overexpressed, which significantly enhanced GA3 production by 250% [258].

In *F. fujikuroi*, the simultaneous deletion of the bikaverin and fusarubin BGCs resulted in enhanced production of GA3, with the Δ*BIK*Δ*FSR* strain showing a 31.67% increase compared to the wild type. Furthermore, the absence of these SM pathways was associated with improved mycelial growth and more efficient carbon utilization, suggesting a reallocation of metabolic resources toward primary growth and GA3 [253].

*F. sporotrichioides*, the causative agent of cereal plant disease *Fusarium* head blight, could produce mycotoxins including trichothecenes and T-2 toxin. 7-Hydroxyisotrichodermin was a shunt pathway metabolite of *F. graminearum*. If 7-hydroxyisotrichodermin was utilized in a cross-species feeding experiment with a trichodiene synthase-deficient mutant of *F. sporotrichioides*, leading to the production of 7-hydroxy T-2 toxin as the end product. When evaluated for cytotoxicity in HL-60 cells, 7-hydroxy T-2 toxin exhibited a potency that was ten-fold lower than that of the parent compound, T-2 toxin [252].

### 7.2. Regulation of Fusarium Co-Cultivation with Other Microorganisms

*F. oxysporum* and *F. fujikuroi* were *Fusarium* species complexes isolated from the nails of patients suffering from onychomycosis. The pure cultures of two *Fusarium* strains only produced trace amounts of fusaric acid. When they were grown in a co-cultivation system, large amounts of fusaric acid were produced. It indicated that there was a regulation between two fungal species [259].

The yield of BEA by *F. oxysporum* AB2 under solid-state fermentation was 22.8 mg/L. When *F. oxysporum* AB2 was co-cultured with another fungus *Epicoccum nigrum* TORT, BEA yield was greatly improved and reached 84.6 mg/L [260].

Two depsipeptides, namely subenniatins A and B, were induced to be produced by co-culturing *F. tricinctum* and *F. begonia*. Neither depsipeptides were observed when either of the two fungal species was cultured alone [261].

The co-cultivation of *F. tritinctum* with the bacterium *Bacillus subtilis* on solid rice medium resulted in the production of nine SMs including macrocarpon C, (−)-citreoisocoumarin, 2-(carboxymethylamino)benzoic acid, (−)-citreoisocoumarinol, lateropyrone, enniatin type cyclic depsipeptides enniatins B, B1 and A1, and lipopeptide fusaristatin A. These metabolites displayed strong antibacterial activity on *B. subtilis*, *Staphylococcus aureus*, *Streptococcus pneumoniae*, and *Enterococcus faecalis*, with MIC values ranging from 2 to 8 μg/mL [262]. Another example was the co-cultivation of *F. tritinctum* with S*treptomyces lividans.* Four new dimeric naphthoquinones, fusatricinones A–D, and a new lateropyrone derivative, dihydrolateropyrone were induced in production of the co-culture system. In addition, several known metabolites such as enniatin derivatives, showed an enhanced accumulation in the co-cultures [263].

### 7.3. Regulation of Transporters

*ZRA1*, a putative ABC transporter gene, was required for zearalenone (ZEA) production in *F. graminearum*. Deletion of *ZRA1* resulted in reduced ZEA production, which indicated that the ABC transporter gene *ZRA1* positively regulated ZEA production [254].

PTR2s were peptide transporters. Three deletion mutants including Δ*FgPTR2A*, Δ*FgPTR2C*, and Δ*FgPTR2D* led to the higher synthesis of DON and ZEA and a reduced synthesis of fusarielin H compared to the wild-type strain of *F. graminearum*. The development of perithecium was actually decreased in these mutants, but not affected by the deletion of *FgPTR2B*. This indicated that PTR2 peptide transporters in *F. graminearum* influenced both sexual development and SM production [264].

### 7.4. Regulation of Development-Related Proteins

Kex2 was a kexin-like protease located in the Golgi apparatus that plays a key role in processing and activating precursor proteins. FvKex2 was required for the fungal normal vegetative growth in *F. verticillioides*. The ∆*Fvkex2* mutant showed a reduced production of FB1 as well as a pathogenicity reduction compared to the wild-type and genetically complemented strains. It indicated that FvKex2 was required for development, virulence, and FB1 production in *F. verticillioides* [265].

### 7.5. Others

*F. fujikuroi* was capable of synthesizing a range of SMs derived from polyketides and non-ribosomal peptides, including fusarins, fusarubins, and bikaverins. The biosynthesis of these compounds relied on key enzymes such as polyketide synthases (PKSs) and non-ribosomal peptide synthetases (NRPSs), which typically required post-translational activation. This modification was mediated by an Sfp-type 4′-phosphopantetheinyl transferase. The *F. fujikuroi* Sfp-type PPTase FfPpt1 was essentially involved in lysine biosynthesis and production of bikaverins, fusarubins and fusarins. The Δ*Ffppt1* mutants revealed an increased production of terpenoid-derived like GAs and volatile compounds such as α-acorenol, which indicated that FfPpt1 negatively regulated the biosynthesis of terpenoids in *F. fujikuroi* [266].

FfCOX17 is a copper chaperone protein in *F. fujikuroi*. The fumonisin production in the ∆*FfCOX17* mutant was significantly increased compared to the WT strain, but the pathogenicity of the ∆*FfCOX17* mutant was not affected, which might be caused by that there was no significant change in the content of gibberellin [267].

HmbC belonged to the high-mobility group (HMG) family to participate in the regulation of carotenoid biosynthesis in *F. fujikuroi*. Deletion of the gene *hmbC* resulted in enhanced carotenoid accumulation and upregulated mRNA expression of genes involved in carotenoid biosynthesis [268].

Non-histone proteins belonging to the high-mobility group (HMG) family are essential structural components of chromatin and contributed to a variety of cellular processes in eukaryotes. In the plant-pathogenic fungus *F. graminearum*, the HMG protein FgNhp6 has been identified as a key regulator influencing pathogenicity, the production of DON, and overall fungal development. The Δ*FgNhp6* deletion mutant showed markedly decreased ability to infect wheat tissues, including coleoptiles and floral spikes, indicating impaired virulence. Surprisingly, despite reduced pathogenicity, these mutants produced higher levels of DON. Genome-wide transcriptome profiling using RNA-seq demonstrated that disruption of *FgNhp6* altered the expression of genes across multiple metabolic networks. Notably, pathways linked to secondary metabolism, such as those governing the biosynthesis of sterols and the pigment aurofusarin, were significantly suppressed in the mutant strain [269].

Both FgWhi2 and FgPsr1 are the stress regulators and phosphatases of *F. graminearum*. They played crucial roles in the regulation of ergosterol and DON biosynthesis, and the response to fungicides in *F. graminearum*. The knockout mutants including Δ*Fgwhi2*, Δ*Fgpsr1*, and Δ*Fgwhi2*Δ*Fgpsr1* all reduced the production of ergosterol and DON, and increased the fungicide sensitivity which positively regulated the sensitivity of *F. graminearum* to fungicides (i.e., chlorothalonil, fluazinam, azoxystrobin, phenamacril, and oligomycin) [270].

Malate synthase is a crucial enzyme in the glyoxylate cycle, which was a supplementary pathway that enabled certain organisms, including many fungi, to synthesize malate from glyoxylate and acetyl-CoA. The deletion of the malate synthase gene *FgMS* in *F. graminearum* reduced mycelial growth rate, decreased sporulation, weakened spore germination, diminished virulence, lowered DON production, increased sensitivity to cell wall stress, and reduced sensitivity to the fungicides including carbendazim, pydiflumetofen, difenoconazole, tebuconazole and phenamacril [271].

In *F. graminearum*, the MAP kinase MGV1 and the transcription factor Tri6 jointly modulated the biosynthesis of DON and fusaoctaxins. This cooperative regulation suggested that MGV1 acted as a central signaling node, enabling selective control over multiple BGCs. Meanwhile, Tri6 was located within a specific BGC, targeted regulatory precision, and controlled the expression of genes within its resident cluster [272].

*FvFUG1* is an unknown gene that has shown a role in pathogenicity and fumonisin biosynthesis in *F. verticillioides*. The fumonisin production was decreased in the Δ*FvFUG1* mutant. Furthermore, the biosynthesis of DIBOA and DIMBOA in maize kernels was also decreased in the Δ*FvFUG1* mutant. FUG1 was considered a novel fungal transcription factor or involved in signal transduction, which needed further confirmation [134].

In *F. verticillioides*, the vacuole and mitochondria patch (vCLAMP) component FvVam6 has been linked to both fungal development and the biosynthesis of FB1 through its role in regulating vacuolar structure. Disruption of the *FvVam6* gene resulted in altered vacuole morphology, indicating its importance in maintaining organelle integrity. Additionally, the Δ*FvVam6* mutant exhibited a substantial decrease in FB1 production, underscoring the connection between proper vacuolar organization and mycotoxin synthesis [254]. Some examples of these regulatory factors to regulate SM production in *Fusarium* fungi are shown in Table 10.

## 8. Discussion

In the early 2000s, the studies on secondary metabolite production through biosynthesis regulation on *Fusarium* fungi were focused on the characterization of single biosynthesis genes and the effects of environmental factors. Currently, the researches were focused more on the regulation of SM BGCs related to a systemic and multifactorial perspective in *Fusarium* fungi. In the past, *Fusarium* secondary metabolites were mainly obtained from plant pathogenic *Fusarium* species. The secondary metabolites usually belonged to mycotoxins or phytotoxins. However, in recent years, more and more SMs were isolated from plant endophytic, soil-derived, and marine-derived *Fusarium* species. In addition to phytotoxic and mycotoxic activities, the biological activities of *Fusarum* SMs are also manifested in many other aspects such as anti-virus, antimalarial, anti-inflammatory, and neuroprotective activities [9].

Many *Fusarium* fungi have been known to produce structurally diverse SMs with a wide range of biological activities that make them a treasure trove of bioactive compounds [273,274,275,276,277,278,279,280,281,282]. To harness the potential of these beneficial SMs, the strategies of biosynthesis regulation essential to activate secondary metabolic pathways, thereby boosting the production of valuable metabolites [283].

The typical example was GA3, a phytohormone synthesized by *F. fujikuroi,* which plays a key role in enhancing crop yield. Despite its agricultural importance, large scale GA3 production has struggled to meet market demand, largely due to insufficient optimization of fermentation conditions and intricate regulatory network governing its biosynthesis. Consequently, improving GA3 yield through targeted regulatory strategies has emerged as an innovative and high-priority approach [40]. Another notable metabolite is bikaverin, a red polyketide pigment produced *F. fujikuroi* and related species which exhibits antiprotozoal and antifungal activities to display its potential as an antibiotic [284]. It is worth noting that an increasing number of *Fusarium*-derived SMs have demonstrated phytotoxic activities [285,286]. These phytotoxins serve as promising lead compounds for the development of novel herbicides, which are the structural analogs synthesized based on these natural scaffolds to show strong herbicidal activity against various weed species [287,288,289,290]. Additionally, acadesine (AICAR) is a compound currently in phase III clinical trials as an anti-tumor agent produced by the endophytic fungus *F. solani*. Its biosynthesis was mediated by the global transcriptional factor VeA, which regulated adenylosuccinate lyase to facilitate acadesine production in *F. solani* [291].

The cryptic metabolite BGCs in fungi can be unlocked through biosynthesis regulation strategies [292]. This approach has successfully led to the discovery of new bioactive metabolites (e.g., dihydrolucilactaene, sansalvamide, apicidins, and fusarielins) from some *Fusarium* species [87,144,237,241,281,293].

For example, treatment of *Fusarium* sp. RK97-94 with the epigenetic modifier NPD938, induced the production of dihydrolucilactaene, a newly identified metabolite exhibiting potent antimalarial activity [241]. Another example was that three new cyclic tetrapeptides apicidins F, J and K (APF, APJ and APK) were produced in the deletion mutants and WT strain of rice pathogen *F. fujikuroi* under conditions of higher nitrogen and acidic pH in a manner dependent on two global regulators, which were nitrogen regulator AreB, and the pH regulator PacC. The BGC of apicidins was named as APF. In addition, over-expression of the atypical pathway-specific TF-encoding gene *APF2* led to the elevated expression of cluster genes under inducing and even repressing conditions and to significantly increase yields of apicidins F, J and K. Among the three cyclic tetrapeptides, apicidin F showed the strongest cytotoxicity and anti-tumor potential [293].

Some examples of the metabolite discovery from *Fusarium* fungi by unlocking the cryptic BGCs through biosynthesis regulation are shown in Table 11.

For the toxic and harmful *Fusarium* SMs, the strategies of biosynthesis regulation should be applied to minimize secondary metabolism to decrease metabolite production [175,294]. These toxic *Fusarium* SMs are usually called mycotoxins, mainly include enniatins, fumonisins, fusaric acid, fusariumic acids, trichothecenes, and zearalenone.

Multiple strategies of biosynthetic regulation could be used to enhance *Fusarium* SM production. These regulation strategies include the OSMAC (one-strain-many compounds) approach [38], co-cultivation of microorganisms [259,262], epigenetic regulation [21,22,295], transcriptional regulation [20], signaling pathway regulation [216], and metabolic shunting [256,273,296,297,298], which have been proven to be effective in promoting the production of fungal SMs.

To reveal more quantities of SMs in *Fusarium* fungi, beyond biosynthesis regulation, other strategies may also be applied including heterologous expression of BGCs [299,300,301], promoter engineering [101,302,303], and combinatorial metabolic engineering [304,305] for either activating *Fusarium* silent BGCs or utilizing some key genes involved in biosynthesis of SMs to mine more metabolites from *Fusarium* fungi. Some examples of the toxic SMs from *Fusarium* fungi are shown in Table 12.

## 9. Conclusions and Perspectives

**Figure 1:**
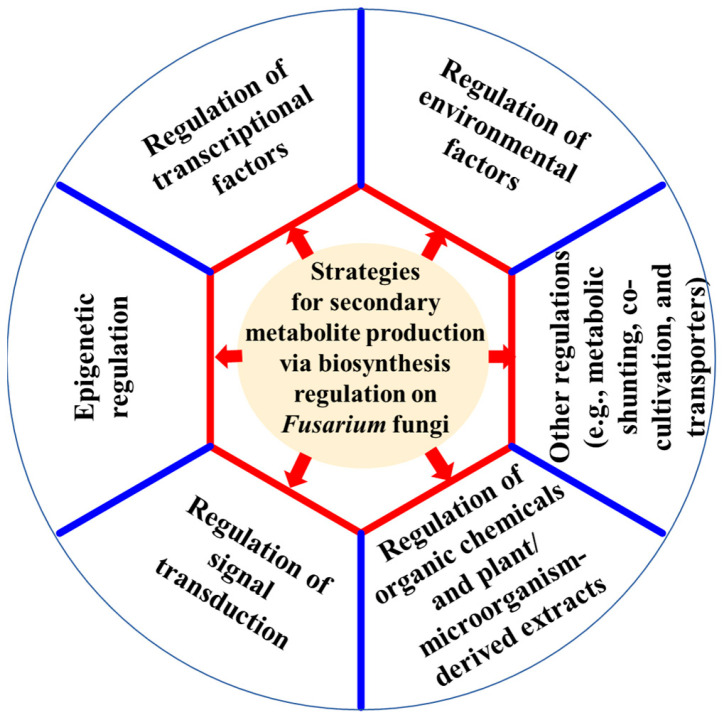
Strategies for SM production via biosynthesis regulation on *Fusarium* fungi.

In summary, *Fusarium* fungi, including marine-derived, soil-derived, endophytic and pathogenic species, can produce large amounts of SMs with a wide range of biological activities to make them a treasure trove of bioactive natural compounds with potential applications. Numerous valuable SMs have been successfully identified from *Fusarium* fungi by using strategies of biosynthesis regulation. The biosynthesis regulation has been considered an effective approach to reveal *Fusarium* metabolites. The strategies to regulate *Fusarium* secondary metabolism mainly include environmental factor regulation, transcriptional factor regulation, epigenetic regulation, and signal transduction regulation (Figure 1). However, *Fusarium* SM production through biosynthesis regulation at multiple levels is a complex process governed by environmental factors together with the complex signaling and regulatory networks involving primary and secondary metabolism in fungi. Most of these networks and their regulatory mechanisms remain unclear.

Overall, the biosynthesis regulation for the production of SMs in *Fusarium* fungi is an efficient strategy for either activating cryptic BGCs and discovering new bioactive metabolites, or increasing the production of low-yield SMs. Furthermore, it can inhibit specific BGCs and decrease toxic metabolite production. The significant efforts will be needed to address the complex regulatory mechanisms of SM biosynthesis in *Fusarium* fungi in future investigations, which may help us better manage the production of *Fusarium* SMs.

## Figures and Tables

**Table 1:** Some examples of LaeA and LaeB regulating SM production in *Fusarium* fungi.

Fungus	Overexpression/ Deletion of *laeA* or *laeB*	Positive/Negative Regulation	Production of SM	Ref.
*F. fujikuroi*	Deletion of *laeA*	Negative	Increased production of bikaverin.	[102]
*F. fujikuroi*	Deletion of *laeA*	Positive	Decreased production of gibberellins A3 and A4, fusarin C, fumonisins B1, B2, B3 and B4, DON, and 15-AcDON.	[102]
*F. fujikuroi*	Deletion of *laeA*	Positive	Decreased production of fusaric acid, fusarinolic acid, and dehydrofusaric acid.	[103]
*F. fujikuroi*	Deletion and overexpression of *laeA*	Positive	Deletion of *laeA* led to decreased production of gibberellins, fumonisins and fusarin C. Overexpression of *laeA* led to increased production of gibberellins.	[104]
*F. fujikuroi*	Deletion of *laeA*	Negative	Increased production of gibepyrones A, B, C, D, E, and F	[105]
*F. graminearum*	Deletion and overexpression of *laeA*	Positive	Deletion of *FglaeA* led to a dramatic reduction in production of trichothecenes and zearalenone. Overexpression of *FglaeA* caused the increased production of trichothecenes and zearalenone.	[28]
*F. oxysporum*	Deletion of *laeA*	Positive	Decreased production of BEA and FA	[106]
*F. oxysporum* f. sp. *niveum*	Deletion of *laeA*	Positive	Decreased production of bikaverin and FA	[29]
*F. verticillioides*	Deletion of *laeA*	Positive	Decreased production of bikaverin, FA, fusarin C and fumonisins.	[107]
*F. pseudograminearum*	Deletion of *laeB*	Positive	Decreased production of DON in the Δ*FpLaeB* mutant	[110]

**Table 2:** Some examples of VeA, VelB and VelC regulating SM production in *Fusarium* fungi.

Fungus	Overexpression/ Deletion of *veA* or *velB*	Positive/Negative Regulation	Production of SM	Ref.
*F. fujikuroi*	Deletion of *veA*	Negative	Increased production of bikaverin.	[102]
*F. fujikuroi*	Deletion of *veA*	Positive	Decreased production of gibberellins A3 and A4, fusarin C, fumonisins B1, B2, B3 and B4, DON, and 15-AcDON.	[102]
*F. fujikuroi*	Deletion of *veA*	Positive	Decreased production of fusaric acid, fusarinolic acid, and dehydrofusaric acid.	[103]
*F. fujikuroi*	Deletion of *veA*	Positive	Decreased production of gibberellins, fumonisins and fusarin C.	[104]
*F. fujikuroi*	Deletion of *veA*	Negative	Increased production of gibepyrones A, B, C, D, E, and F.	[105]
*F. graminearium*	Deletion of *veA*	Positive	Reduced production of DON.	[111]
*F. graminearium*	Deletion of *veA*	Positive	Decreased production of trichothecenes.	[112]
*F. nematophilum*	Overexpression of *veA*	Positive	Increased production of antitumor compounds.	[113]
*F. oxysporum*	Deletion of *veA*	Positive	Decreased production of BEA and FA.	[106]
*F. oxysporum* f. sp. *niveum*	Deletion of *veA*	Positive	Decreased production of bikaverin and FA.	[29]
*F. solani*	Overexpression of *veA*	Positive	Increased production of antitumor substances	[114]
*F. solani*	Deletion of *veA*	Negative	Increased production of acadesine	[115]
*F. verticillioides*	Deletion of *veA*	Positive	Decreased production of fusarin C and fumonisins B1, B2 and B3.	[116]
*F. verticillioides*	Deletion of *veA*	Positive	Decreased production of fumonisins.	[117]
*F. fujikuroi*	Deletion of *velB*	Positive	Decreased production of gibberellins, fumonisins and fusarin C.	[104]
*F. fujikuroi*	Deletion of *velB*	Negative	Increased production of gibepyrones A, B, C, D, E, and F	[105]
*F. graminearum*	Deletion of *velB*	Positive	Decreased production of DON.	[118]
*F. graminearum*	Deletion of *velB*	Positive	Decreased production of trichothecenes and ZEN.	[119]
*F. proliferatum*	Deletion of *velC*	Negative	Enhanced production of FB1 and FA	[120]
*F. pseudograminearum*	Deletion of *velB*	Positive	Decreased production of DON.	[33]

**Table 3:** Some examples of AreA and AreB regulating SM production in *Fusarium* fungi.

Fungus	Overexpression/ Deletion of *areA* and *areB*	Positive/Negative Regulation	Production of SM	Ref.
*F. graminearum*	Deletion of *areA*	Positive	Decreased production of DON	[128]
*F. graminearum*	Deletion of *areA*	Positive	Decreased production of trichothecene biosynthesis	[129]
*F. graminearum*	Overexpression of *areA*	Positive	Increased production of gibberellins and bikaverin	[130]
*F. oxysporum*	Deletion of *areA*	Positive	Decreased production of ferricrocin and BEA	[131]
*F. proliferatum*	Deletion of *areA*	Positive	Significantly decreased production of fumonisin	[132]
*F. verticillioides*	Deletion of *areA*	Positive	Lack of FB1 production	[133]

**Table 4:** Some examples of pathway-specific transcriptional factors regulating SM production in *Fusarium* fungi.

Fungus	Overexpression/ Deletion of the Pathway-Specific TF Gene	Positive/Negative Regulation	Production of SM	Ref.
*F. fujikuroi*	Overexpression of *Fum21*	Positive	Strongly activated the FUM cluster genes leading to 1000-fold elevated FBx levels.	[141]
*F. fujikuroi*	Deletion of *Fub10*	Positive	Decreased production of FA	[142]
*F. graminearum*	Deletion of *GIP2*	Positive	Decreased production of aurofusarin	[143]
*F. graminearum*	Overexpression of *GIP2*	Positive	Increased production of aurofusarin	[143]
*F. graminearum*	Activation of FSL	Positive	Induced production of fusarielins F, G and H	[144]
*F. graminearum*	Deletion of *Tri6*	Positive	Reduced production of trichothecenes	[145]
*F. graminearum*	Deletion of *Tri10*	Positive	Reduced production of trichothecenes	[145]
*F. graminearum*	Activation of ZEB2	Positive	Increased production of ZEN	[150]
*F. verticillioides*	Deletion of *fum21*	Positive	Decreased production of fumonisins	[151]

**Table 5:** Some examples of the miscellaneous transcriptional factors regulating SM production in *Fusarium* fungi.

Fungus	Overexpression/ Deletion of the TF Gene	Positive/Negative Regulation	Production of SM	Ref.
*F. avenaceum*	Overexpression of *esyn1*	Positive	Increased production of ENs.	[24]
*F. fujikuroi*	Deletion of *meaBL* and *meaBS*	Negative	Increased production of GAs	[153]
*F. fujikuroi*	Overexpression of *FfSge1*	Positive	Increased production of FUM, FU, and APF in the *FfSge1*-OE strain	[155]
*F. verticillioides*	Deletion of *sge1*	Positive	Decreased production of fumonisins.	[156]
*F. verticillioides*	Deletion of *art1*	Positive	Decreased production of fumonisins including FB1	[157]
*F. graminearum*	Deletion of *Fgart1*	Positive	Decreased production of trichothecenes	[157]
*F. graminearum*	Deletion of *FgStuA*	Positive	Reduced production of DON and 15-AcDON	[158]
*F. oxysprum* f. sp. *cubense*	Deletion of *FoAce2*	Positive	Decreased production of BEA.	[160]
*F. oxysporum* f. sp. *lycopersici*	Deletion of *FolCzf1*	Positive	Reduced production of FA	[163]
*F. pseudograminearum*	Deletion of *Fp487*	Positive	Reduced production of 3-AcDON	[164]
*F. verticillioides*	Deletion and overexpression of *zfr1*	Positive	Positive regulation of fumonisin production	[165]
*F. verticillioides*	Deletion of *HAP3*	Positive	Suppressed production of fumonisins.	[166]
*F. verticillioides*	Deletion of *hxk1*	Positive	Decreased production of trehalose and FB1	[167]
*F. verticillioides*	Deletion of *FvatfA*	Negative	Overproduction of bikaverin in the Δ*FvatfA* strain	[168]
*F. verticillioides*	Deletion of *FvatfA*	Positive	Abolishment of fumonisin production in the Δ*FvatfA* strain	[168]
*F. verticillioides*	Deletion of *MADS1* and *MADS2*	Positive	Decreased production of FB1	[169]
*F. verticillioides*	Deletion of *FvOshC*	Positive	Decreased production of FB1	[170]
*Fusarium* sp.	Deletion of *Fsptf*	Positive	Decreased production of javanicin and fusarubin	[171]

**Table 6:** Some examples of HATs regulating SM production in *Fusarium* fungi.

Fungus	Overexpression/ Deletion of the HAT Gene	Positive/Negative Regulation	Production of SM	Ref.
*F. graminearum*	Deletion of *elp3*	Negative	Decreased production of trichothecenes such as DON and 15-AcDON	[177]
*F. fujikuroi*	Deletion of *GcnE*	Positive	Decreased production of 18 metabolites	[179]
*F. graminearum*	Deletion of *gcn5*	Positive	Decreased production of DON.	[180]
*F. graminearum*	Deletion of *gcn5*	Positive	Inhibited production of DON.	[181]
*F. graminearum*	Deletion of *gcn5*	Positive	Inhibited production of DON.	[182]
*F. graminearum*	Deletion of the bromodomain of *FgGCN5*	Positive	Significant reduction in DON production	[183]
*F. fujikuroi*	Deletion of *hat1*	Positive	Decreased GA production.	[104]
*F. fujikuroi*	Overexpression of *hat1*	Positive	Increased GA production.	[104]
*F. graminearum*	Deletion of *sas3*	Positive	Decreased production of DON.	[178]
*F. graminearum*	Deletion of *hat1*	Positive	Severe defects in DON production	[184]
*F. graminearum*	Deletion of *hat2*	Positive	Decreased production of DON	[185]

**Table 7:** Some examples of HDACs regulating SM production in *Fusarium* fungi.

Fungus	Overexpression/ Deletion of the HDAC Gene	Positive/Negative Regulation	Production of SM	Ref.
**Overexpression** **/Deletion of Class I HDAC Gene**				
*F. verticillioides*	Deletion of *hos2*	Positive	Decreased FB1 production.	[35]
*F. verticillioides*	Overexpression of *rpd3*	Positive	Increased FB1 production.	[35]
*F. fujikuroi*	Deletion of *hda2*	Positive	Decreased production of GA3, GA4, GA7, BIK, FSR, and FU	[186]
**Overexpression/Deletion of Class II HDAC Gene**				
*F. fujikuroi*	Deletion of *hda1*	Positive	Decreased production of GA3, GA4, GA7, bikaverin, fusarubin, and FA	[186]
*F. fujikuroi*	Deletion of *hda1*	Negative	Increased production of fusarin A	[186]
*F. fujikuroi*	Deletion of *hda1*	Negative	Increased production of BEA	[187]
*F. verticillioides*	Deletion of *hda1*	Negative	Increased production of FB1	[35]
*F. graminearum*	Deletion of *hdf1*	Positive	Decreased production of DON	[188]
*F. asiaticum*	Deletion of *hdf2*	Negative	Increased production of 4-ANIV and 4,15-diAcNIV.	[189]
**Overexpression/Deletion of Class III HDAC Gene**				
*F. verticillioides*	Deletion of *hst2*	Negative	Increased production of FB1	[35]
*F. verticillioides*	Overexpression of *sirt4*	Negative	Decreased production of FB1	[35]
*F. verticillioides*	Deletion of *sir2*	Negative	Increased production of FB1	[35]
*F. verticillioides*	Deletion of *sirt5*	Positive	Decreased production of FB1	[191]
*F. verticillioides*	Deletion of *sir2*	Positive	Decreased production of FB1	[191]

**Table 8:** Some examples of HMTs regulating SM production in *Fusarium* fungi.

Fungus	Overexpression/ Deletion of the HMT Gene	Positive/Negative Regulation	Production of SM	Ref.
*F. graminearum*	Deletion of *kmt6*	Negative	Increased production of SMs	[194]
*F. fujikuroi*	Deletion of *Ccl1*	Positive	Decreased production of GA3	[195]
*F. fujikuroi*	Deletion of *set2* and *ash1*	Positive	Decreased production of SMs	[196]
*F. fujikuroi*	Deletion of *kmt5*	Positive	Decreased production of fusarin C	[197]
*F. graminearum*	Deletion of *Ccl1*	Positive	Decreased production of DON	[195]
*F. graminearum*	Deletion	Positive	Decreased production of ZEN	[197]
*F. graminearum*	Deletion of *FgSet1*	Positive	Decreased production of DON and aurofusarin	[199]
*F. graminearum*	Deletion of *kmt6*	Negative	Led to the production of fusaristain A	[200]
*F. graminearum*	Overexpression of *kmt6*	Positive	Led to the production of three pyrone derivatives gibepyrone A, and fusapyrones A and B	[200]
*F. mangiferae*	Deletion of *Fmkmt1*	Positive	An almost complete loss of fusapyrone and deoxyfusapyone	[201]
*F. proliferatum*	Deletion of *Fpkmt1*	Negative	Increased production of GAs	[32]
*F. verticillioides*	Deletion of *FvSet1*	Positive	Significant defect in FB1 biosynthesis.	[202]

**Table 9:** Some examples of chemicals to regulate SM production in *Fusarium* fungi.

Fungus	Chemical and Its Concentration	Production of SM	Ref.
*F. asiaticum*	Agmatine (2 mM)	Increased production of GABA and DON.	[224]
*F. culmorum*	Compactin (25 μg/mL)	Suppressed production of DON and ZEN.	[226]
*F. graminearum*	6PAP (62.5 μg/mL)	Reduced production of DON	[227]
*F. graminearum*	Pinoresinol (1.25, 5.0 mg/L)	Decreased production of DON and NIV	[228]
*F. graminearum*	Secoisolariciresinol (1.25, 5.0 mg/L)	Decreased production of DON and NIV	[228]
*F. graminearum*	Caffeic acid (0.5 mM)	Inhibited production of DON.	[229]
*F. graminearum*	NPD12671 (2 μM)	Stimulated production of 15-AcDON	[230]
*F. graminearum*	DHA (5 μM)	Inhibited production of 15-AcDON	[230]
*F. graminearum*	Nicotinamide (500 μM)	Decreased production of ergosterol peroxide and DON.	[231]
*F. graminearum*	TSA (10 μg/mL)	An increase in trichodiene synthase gene (*Tri5*) expression	[232]
*F. graminearum*	Phenazine-1-carboxamide	Decreased DON production by inhibiting FgGcn5.	[233]
*F. graminearum*	2-Hydroxy-4-methoxybenzaldehyde (100 μg/mL)	Reduced production of ergosterol and DON.	[234]
*F. graminearum*	Citric acid (5 or 10 mM)	Decreased production of type B trichothecenes	[235]
*F. oxysporum*	Prednisone (300 μM)	Increased production of umbelliferone	[236]
*F. oxysporum* f. sp. *conglutinans*	SBHA (500 μM)	Induced production of two FA derivatives namely 5-butyl-6-oxo-1,6-dihydropyridine-2-carboxylic acid and 5-(but-9-enyl)-6-oxo-1,6-dihydropyridine-2-carboxylic acid.	[237]
*F. verticillioides*	5-Azacytidine (25 μM)	5-Azacytidine had some impacts on these metabolites.	[238]
*F. verticillioides*	Sodium valproate (100 μM)	Induced the alteration of the metabolic profile by promoting the expression of cryptic genes.	[238]
*F. verticillioides*	Di-hydroxyoctadecenoic acids.	Reduced production of fumonisins	[239]
*Fusarium* sp. RK97-94	Hygromycin B (100 μg/mL)	Induced the production of SMs, including lucilactaene, NG-391, fusarubin, 1233A, and 1233B, in *Fusarium* sp. RK97-94.	[240]
*Fusarium* sp. RK97-94	NPD938 (30 μM)	Induced production of three lucilactaene analogures, namely dihydroNG391, dihydrolucilactaene, and 13α-hydroxylucilactaene.	[241]

**Table 10:** Some examples of other regulatory factors to regulate SM production in *Fusarium* fungi.

Fungus	Overexpression/ Deletion of Gene	Positive/Negative Regulation	Production of SM	Ref.
*F. fujikuroi*	Deletion of *FgCOX17*	Negative	Significant increase in fumonisin production	[267]
*F. fujikuroi*	Deletion of *hmbC*	Negative	Increased production of carotenoids	[268]
*F. fujikuroi*	Deletion of the bikaverin and fusarubin biosynthesis gene clusters	Negative	Significant increase in GA3 production	[253]
*F. graminearum*	Deletion of *FgNhp6*	Negative	Significant increase in DON production	[269]
*F. graminearum*	Deletion of *Fgwhi2*	Positive	Significant decrease in DON and ergosterol production	[270]
*F. graminearum*	Deletion of *Fgpsr1*	Positive	Significant decrease in DON and ergosterol production	[270]
*F. graminearum*	Deletion of *FgMS*	Positive	Decreased production of DON	[271]
*F. graminearum*	Deletion of *FgPTR2A, FgPTR2C*, and *FgPTR2D*	Positive	Increased production of DON and zearalenone, and decreased production of fusarielin H	[264]
*F. verticillioides*	Deletion of *FUG1*	Positive	Decreased production of fumonisin, DIMBOA and DIBOA	[134]
*F. verticillioides*	Deletion of *FvVam6*	Positive	Significantly reduced FB1 production	[255]
*F. verticillioides*	Deletion of *Fvkex2*	Positive	Reduced production of FB1	[265]

**Table 11:** Some examples of the metabolite discovery from *Fusarium* fungi by unlocking the cryptic BGCs through biosynthesis regulation.

*Fusarium* Species	New Metabolite	Biological Activity	Biosynthesis Regulation Strategy	Ref.
*F. pseudograminearum*	A novel cytokinin	Plant growth regulation	OSMAC strategy	[87]
*F. graminearum*	Fusarielins F, G and H	Cytotoxic activity	Pathway-specific transcriptional factor regulation	[144]
*F. oxysporum* f. sp. *conglutinans*	5-butyl-6-oxo-1,6-dihydropyridine-2-carboxylic acid and 5-(but-9-enyl)-6-oxo-1,6-dihydropyridine-2-carboxylic acid	Not mentioned	Treatment of epigenetic modifier SBHA	[237]
*Fusarium* sp. RK97-94	Dihydrolucilactaene	Antimalarial activity	Treatment of epigenetic modifier NPD938	[241]
*Fusarium* sp. CNL 292	Sansalvamide	Cytotoxic activity	OSMAC strategy	[281]
*F. fujikuroi*	Apicidins F, J and K	Cytotoxic activity	OSMAC strategy; regulation of global transcriptional factors AreB and PacC, and pathway-specific transcriptional factor APF2	[293]

**Table 12:** Some examples of the toxic SMs from *Fusarium* fungi.

Metabolite	Toxicity	Metabolite-Producing *Fusrium* Species	Ref.
Fumonisins	Causing oesophageal cancer and neural tube defects; Phytotoxic activity by inhibiting root and shoot growth and causing chlorosis, necrosis, and wilting.	*F. fujikuroi*, *F. oxysporum*, *F. proliferatum*, and *F. verticillioides*	[12]
Enniatins	Causing mitochondrial dysfunction, lysosomal alteration, cell cycle disruption, and lipid peroxidation; Disrupting cell membranes by increasing permeability by forming pore structures.	*F. avenaceum*	[306]
FA, 9,10-dehydrofusaric acid	Phytotoxic activity causes plant wilting.	*F. oxysporum*, *F. moniliforme*, *F. heterosporum*	[307]
MON	Inhibiting protein synthesis, causes chromosome damage.	*F. proliferatum*, *F. fujikuroi*, and *F. nygamai*	[307]
Fusariumic acids	Phytotoxic activity causes plant wilting.	*F. oxysporum* f. sp. *radicis-lycopersici*	[308]
Trichothecenes including HT-2 toxin, T-2 toxin, DON, NIV, 3-AcDON, and 15-AcDON	Causing hepatotoxicity, enterotoxicity, neurotoxicity, and reproductive toxicity in animals and humans.	*F. graminearum*, *F. sporothichioides*, *F. langsethiae* and *F. culmorum*	[309]
Zearalenone (ZEN), zearalanone (ZAN)	Estrogenic effects on animals and humans; cause genetic toxicity, reproductive toxicity, hepatotoxicity, immunotoxicity, carcinogenicity, and so on.	*F. graminearum*, *F. oxysporum*, *F. equisetum*, *F. nivalis* and *F. sambucinum*	[310]

## Data Availability

No new data were created or analyzed in this study. Data sharing is not applicable to this article.

## Abbreviations

The following abbreviations are used in this manuscript:

3-AcDON	3-acetyl deoxynivalenol
15-AcDON	15-acetyl deoxynivalenol
4-AcNIV	4-acetyl nivalenol
AFB1	aflatoxin B1
APF	apicidin F
15-AS	15-acetoxyscipenol
a_w_	water availability
BEA	Beauvericin
BGC	biosynthetic gene cluster
cAMP	cyclic adenosine monophosphate
COMPASS	Complex Proteins Associated with Set1
DAS	Diacetoxyscirpenol
4,15-diAcNIV	4,15-diacetyl nivalenol
DNMT	DNA methyltransferase
DON	Deoxynivalenol
EN	Enniatin
FA	fusaric acid
FB1	fumonisin B1
FB2	fumonisin B2
FB3	fumonisin B3
FU	fusaric acid
FUM	Fumonisin
GA3	gibberellic acid
GAs	Gibberellins
HAT	histone acetyltransferase
H4K20me	monomethylation of lysine 20 of histone 4
H3K9me3	trimethylation of lysine 9 of histone 3 (hitone 3 lysine 9)
HDAC	histone deacetylase
HDM	Histone demethylase
HMT	histone methyltransferase
MAPK	mitogen-activated protein kinase
MON	Moniliformin
MIC	Minimal inhibitory concentration
NIV	Nivalenol
NRPS	non-ribosomal peptide synthetase
OE	Overexpression
OSMAC	one strain many compounds
6-PAP	6-pentyl-α-pyrone
PDA	potato dextrose agar
PKS	polyketide synthase
PRC2	polycomb repressive complex 2
PTM	post-translational modification
SAM	*S*-adenocyl-1-methionine
SBHA	suberoyl bishydroxamic acid
SM	secondary metabolite
T-2 toxin	fusariotoxin T2
TF	transcriptional (regulatory) factor
TOR	target of rapamycin
WT	wild-type
ZEN, ZEA	Zearalenone
